# Combined treatment with anti-PSMA antibody and human peripheral blood-derived NK cells for castration-resistant prostate cancer

**DOI:** 10.3389/fimmu.2025.1572676

**Published:** 2025-05-21

**Authors:** Fangming Wang, Nianzeng Xing, Jianxing Li

**Affiliations:** ^1^ Department of Urology, Beijing Tsinghua Changgung Hospital, School of Clinical Medicine, Tsinghua University, Beijing, China; ^2^ Department of Urology, National Cancer Center/National Clinical Research Center for Cancer/Cancer Hospital, Chinese Academy of Medical Sciences and Peking Union Medical College, Beijing, China

**Keywords:** immunotherapy, prostate-specific membrane antigen, antibody, natural killer cells, castration-resistant prostate cancer

## Abstract

**Background:**

Castration-resistant prostate cancer (CRPC) has a poor prognosis and requires novel therapeutic approaches. Previously, we discovered that a high dose of human peripheral blood-derived natural killer (PB-NK) cells can have antitumor effects against CRPC. However, whether antibodies against prostate-specific membrane antigen (PSMA) can direct adoptive NK cells to the tumor site and therefore decrease NK cell dosage through antibody-dependent cellular cytotoxicity remains unknown.

**Methods:**

NK cells were obtained from the blood samples of healthy donors. To engineer an anti-PSMA antibody (Ab), a llama was immunized with human PSMA protein, and the anti-PSMA variable domains of camelid heavy-chain antibody (VHH) clones were isolated using phage display. The VHH was recombinantly fused with the human Fc region to produce an anti-PSMA Ab. *In vitro*, NK cell cytotoxicity was evaluated using cell counting kit-8. Levels of cytokines and prostate-specific antigen (PSA) were determined using ELISA. The expression of CD107a and CD16 (the Ab Fc-receptor) in NK cells and the Ab affinity were detected using flow cytometry. Antitumor effects were evaluated in patient-derived organoid (PDO) models and in 22RV1 tumor-bearing mice *in vivo*.

**Results:**

We constructed an anti-PSMA Ab and validated its high affinity toward the PSMA antigen. CD16 is abundantly expressed in PB-NK cells. The anti-PSMA Ab significantly enhanced the cytotoxicity of NK cells against CRPC cells *in vitro*, evidenced by increased killing rate, upregulation of the degranulation marker CD107a, increased secretion of interferon-γ, and decreased PSA levels. Furthermore, our combined treatment showed powerful antitumor effects in PDO and CRPC xenograft mouse models.

**Conclusion:**

Combined treatment with anti-PSMA Ab and human PB-NK cells improves antitumor efficacy against CRPC and is a promising approach to treating CRPC in clinical settings.

## Introduction

Prostate cancer (PCa) is the most common cancer in men in 112 countries, and the annual number of new cases is projected to rise to 2.9 million by 2040 (1). Early and localized PCa can be controlled by radiation, radical prostatectomy (RP), or active surveillance, and androgen deprivation therapy (ADT) is preferable for advanced PCa. However, most cases of PCa eventually develop into castration-resistant prostate cancer (CRPC), which is currently incurable (2). Therefore, novel and efficacious therapeutic strategies are required to prolong the survival of patients with PCa. In this regard, immunotherapies have shown mixed results in the treatment of PCa. The use of sipuleucel-T, which is composed of autologous antigen-presenting cells cultured with a fusion protein, has been shown to prolong overall survival among men with metastatic CRPC (3). However, sipuleucel-T has a high cost (currently over $100,000) and is limited due to its autologous nature (4). Immune checkpoint inhibitors (ICI) targeting PD-1/PD-L1 and CTLA-4 have shown substantially lower response rates in PCa than in other solid tumors due to the cold and immunosuppressive tumor microenvironment (TME) (5, 6). Among several options of immune effectors, natural killer (NK) cells have the advantage of not causing graft-versus-host disease, allowing off-the-shelf administration, exhibiting few toxicities, such as cytokine release syndrome (CRS) or neurotoxicity, and exhibiting a short period survival time in the host when comparing T cells (7–10). For these reasons, our team has been dedicated to NK cell therapy for CRPC in recent years (11–13). Previously, we showed that allogeneic peripheral blood-derived NK (PB-NK) cells exert anti-tumor effects against CRPC (11). However, this therapy displays high systemic toxicity and low tissue selectivity. There are multiple strategies to potentiate adoptive NK cell responses for therapeutic purposes in malignant tumors, such as the use of chimeric antigen receptor-engineered NK (CAR-NK) cells, bispecific and trispecific killer cell engagers (BiKEs and TriKEs), and checkpoint blockades (14). Based on these strategies, to make NK cells more specifically recognize and kill PCa cells and to reduce the NK cell dosage, we successfully constructed CAR-NK cells targeting the prostate-specific membrane antigen (PSMA), and subsequently demonstrated their specific anti-tumor effects against PCa (13). As the CAR-NK manufacturing process is complicated and may involve safety problems owing to virus transfection, we decided to adopt a strategy of adding a specific antibody towards PSMA based on NK cell therapy.

The NK cell-mediated anti-tumor activity is regulated by an array of activating and inhibitory cell surface receptors. The activating receptors predominantly include CD16, KIR-2DS, KIR-3DS, natural cytotoxicity receptor family members (NKp46, NKp44, and NKp30), NKG2D, 2B4, CD226, and CD94/NKG2C (15). Notably, CD16 is the most potent activating receptor and the only receptor that can independently activate NK cells without any additional activation through other receptors (16, 17). The binding of CD16 to the Fc regions of IgG antibodies on opsonized cells can activate NK cells through a process termed antibody-dependent cell-mediated cytotoxicity (ADCC) (18). Furthermore, the expression of PSMA, an integral non-shed type 2 membrane protein, is high in prostate epithelial cells and is 100–1000 times higher in PCa than in normal tissue (19). Therefore, the anti-PSMA antibody (Ab), redirects NK cells (including innate and adoptive NK cells) to target PCa cells through the tumor-specific target molecule PSMA on the one hand, and to CD16-positive NK cells on the other hand, forming cytolytic synapses and activating NK cells to release cytotoxic granules containing perforin and granzymes to directly lyse PCa cells and cytokines to recruit other immune cells to the tumor site (20).

In the present study, we immunized llamas with recombinant human PSMA protein and PSMA-positive PCa cell lysate to generate anti-PSMA variable domains of camelid heavy-chain antibodies (VHHs). We then screened and identified an anti-PSMA VHH clone with the highest affinity and selectivity using phage display. Next, we recombinantly fused VHH to the human Fc region to produce an anti-PSMA Ab. Finally, we evaluated the therapeutic efficacy of the anti-PSMA Ab and human PB-NK cells against CRPC. We believe that the anti-PSMA Ab can precisely direct adoptive NK cells to PCa tissues and exert potent cytotoxic effects.

## Materials and methods

### Cell lines and culture

Human PCa cell lines (LNCaP, PC3, C4-2, 22RV1, and DU145), a prostate epithelial cell line (RWPE-1), and a bladder cancer (BCa) cell line (T24) were purchased from the American Type Culture Collection. Cancer cell lines were cultured in RPMI-1640 medium (Servicebio) supplemented with 10% fetal bovine serum (FBS) (Biological Industries) with 1% penicillin/streptomycin (Solarbio, China), whereas RWPE-1 cells were cultured in Dulbecco’s modified Eagle’s medium (HyClone) with 10% FBS. Cultured cells were assessed using the TransDetect^®^ PCR Mycoplasma Detection Kit (TransGen Biotech) once monthly, and all tests were negative for mycoplasma.

### Preparation of PB-NK cells

As described previously, PB-NK cells were obtained from the peripheral blood mononuclear cells (PBMCs) of healthy donors (11).

Informed written consent from all participants or next of kin was obtained prior to the research, and this procedure was approved by the Ethics Committee of Beijing Tsinghua Changgung Hospital (ethical approval number: IRB24351-0-01). Five healthy adult donors (including three males and two females) were enrolled in the study. The range was 25–40 years. These PBMCs were cultivated in an OpTmizer CTS T-cell expansion serum-free medium (Invitrogen, Carlsbad, CA, USA) containing human IL-15, IL-2, and OK432 (T&L Biology Technology Co. Ltd.) at 37°C with 5% CO_2_. On day 14, pure NK cells were obtained after CD3 negative and CD56 positive selection using magnetic beads (Miltenyi Biotec). Cell purity and counts were evaluated using flow cytometry, and the fold expansion was calculated using cell counts before and after culture.

### PSMA expression from bioinformatic analysis and PCa specimens

The expression of PSMA (FOLH1 gene) was analyzed with Gene Expression Profiling Interactive Analysis (GEPIA; http://gepia.cancer-pku.cn/) and the Prostate Cancer Transcriptome Atlas (PCTA; http://www.thepcta.org/; choose “PCTA database”) by entering the gene name “FOLH1” and then selecting the tumor type “PRAD” and the analysis content “Gene expression profile” or “by disease course.” Tissue samples for immunohistochemistry (IHC) analysis were collected from patients diagnosed with prostate adenocarcinoma and treated with RP at Beijing Tsinghua Changgung Hospital. Patients who received additional treatment, such as neoadjuvant therapy, radiotherapy, or chemotherapy, were excluded. Briefly, tissue sections were incubated with primary antibodies against PSMA (1:1000, Cell Signaling) at 4°C overnight, followed by counterstaining with hematoxylin, dehydration, and mounting of the slides. The PSMA endothelial cell staining score was semi-quantitatively graded (0+ to 3+) as follows: the percentage of stained cells (0–100%) was multiplied by the corresponding IHC signal staining intensity (0+ to 3+) to obtain the final staining scores, which were categorized as follows: 0+ (negative), 1+ (0–99), 2+ (100–199), and 3+ (200–300). The PCa specimen was divided into 3 groups according to grade: Gleason score ≤ 6; 7; ≥ 8, and the PSMA expression was compared among groups. The pathology of tissue staining was conducted and reviewed independently by 2 senior pathologists from the Department of Pathology, Beijing Tsinghua Changgung Hospital. Before the grading, they received rigorous standardized training and independently evaluated the staining results according to the established scoring criteria. Subsequently, a consistency test was performed on their scoring results to ensure the accuracy and reliability of the grading. In case of any discrepancies, they would discuss together or consult a third more senior pathologist to ensure the accuracy of the evaluation results.

### Flow cytometry

The PCa, BCa, and prostate epithelial cell lines were stained with PE-Cy7 anti-PSMA (BioLegend, LNI-17 clone) to detect PSMA protein expression on cell surfaces. PSMA-positive (LNCaP and 22RV1) and-negative (PC3 and T24) cell lines were stained with anti-PSMA VHHs for 60 min, followed by incubation with APC-conjugated anti-mouse IgG (BioLegend, Poly4053 clone) to validate the affinity and specificity of anti-PSMA VHHs. The anti-human APC-conjugated PSMA mAb (Biolegend, LNI-17 clone) was used as a positive control. Both the LNCaP and PC3 cell lines were stained with anti-PSMA Ab for 60 min, followed by incubation with APC-conjugated anti-human IgG-Fc (Sino Biological, SSA015 clone) to validate the affinity and specificity of the anti-PSMA Ab. The PBMCs or PBMC-derived immune cells were stained with a combination of anti-CD3 (OT3), anti-CD56 (NCAN), and anti-CD16 (3G8) during NK cell culture (antibodies obtained from BioLegend). After co-culturing with or without 22RV1 cells (effector-to-target ratios (E/T) = 1:1) in the presence of IgG control or anti-PSMA Ab for 6 h, NK cells were collected and stained with anti-CD107a (LAMP-1) (BioLegend, H4A3) for the degranulation assay. The cells were also stained with the corresponding isotype antibodies as controls. Data were acquired using a flow cytometer (LSR Fortessa; BD Biosciences) and analyzed using FlowJo software (version 10; Treestar, USA).

### Western blotting analysis

As described previously (13), cancer cells (T24/LNCaP/PC3/C4-2/22RV1/DU145) in the logarithmic growth phase were collected and lysed with RIPA lysis buffer to extract total proteins, and the protein concentration was determined using a BCA protein quantification kit. Then, the protein samples were separated by sodium dodecyl sulfate-polyacrylamide gel electrophoresis (SDS-PAGE) electrophoresis and transferred to a PVDF membrane. Next, the PVDF membrane was blocked with 5% skim milk for 2 hours to reduce non-specific binding. After blocking, anti-PSMA rabbit IgG (1:500; Cell Signaling Technology, D7I8E) and anti-β-actin rabbit IgG (1:5000; ImmunoWay, PT0519R) primary antibodies were added respectively and incubated overnight at 4°C. The next day, the membrane was washed 3 times with TBST for 10 minutes each time, and then the corresponding horse radish peroxidase-labeled secondary antibodies were added and incubated at 25°C for 2 hours. Finally, the bands were illuminated using ECL (Millipore), and the exposed X-ray films were screened. β-actin was used as an internal reference for semi-quantitative analysis of PSMA protein expression levels.

### Cell counting kit-8 assay

A CCK-8 assay was performed according to our previous method (12). Briefly, 22RV1, PC3, and RWPE-1 cells in the logarithmic growth phase were seeded in 96-well plates at 10–000 cells/well. On day two, NK cells were added at two different E/T ratios (E/T = 0.5:1 and 1:1) with anti-PSMA Ab (5, 10, 20 μg/mL) or IgG1 isotype mAb (10 μg/ml) (Med Chem Express). Mixed incubations were performed for 2 and 6 h. The optical density (OD) of each well was measured at 450 nm using an enzyme-linked immunosorbent assay (ELISA) plate reader (Multiskan FC; Thermo FC). The killing rate was calculated as follows: killing rate = (OD_control_ − OD_sample_/OD_control_ − OD_medium_) × 100%.

### Cytokine release and prostate-specific antigen secretion assays

Initially, 1 × 10^6^ 22RV1 cells were plated in a 6-well plate (Corning). After 12 h, 1 × 10^6^ NK cells with or without 10 μg/mL of anti-PSMA Ab or IgG were added to the target cells. NK cells alone were used as controls. The supernatant was collected after centrifugation for 6 h. Concentrations of interferon (IFN)-γ, tumor necrosis factor (TNF)-α, perforin-1, granzyme B, and PSA in the supernatant were measured using human ELISA kits for IFN-γ (Invitrogen), TNF-α (CUSABIO), PRF1/PFP (CUSABIO), granzyme B (CUSABIO), and PSA (CUSABIO), respectively, according to the manufacturer’s instructions. Blood was collected from the inferior vena cava to measure interleukin (IL)-6 levels on day 28 after the mice were euthanized in the subcutaneous tumor model.

### Construction and validation of anti-PSMA Ab

The generation of an anti-human PSMA Ab immune response in llamas has been previously described elsewhere (21). The Ethics Committee of Beijing Tsinghua Changgung Hospital (ethical approval number: IRB24351-0-01) approved all procedures of llama management, inoculation, and sample collection, which were performed in accordance with the Principles of Laboratory Animal Care (NIH publication Vol 25, no. 28 revised 1996). Briefly, a llama was simultaneously immunized with recombinant human PSMA protein and LNCaP cell lysate. After immunization, serum samples were analyzed by ELISA to confirm the generation of an anti-PSMA immune response. Nanobodies were selected by phage display, and library construction was performed as previously reported (22). Briefly, peripheral blood lymphocytes were isolated from the blood of immunized llamas (23), RNA was extracted and reverse-transcribed into cDNA, and VHH genes were amplified by nested PCR. The VHH fragments were ligated into the pComb3XSS-2 phagemid vector and transformed into TG1 Escherichia coli cells to construct a nanobody immune library. Subsequently, the nanobody library was infected with M13 K07 hyperphages to prepare a phage-displayed library, and three rounds of biopanning were carried out. During the biopanning process, the ELISA method was used for negative selection with PC3 cells first and then positive selection with LNCaP cells to select clones with high binding affinity to the PSMA antigen. The selected clones were sequenced, and distinct nanobody clones were identified. The anti-PSMA VHH (1H5) was recombinantly fused with the human IgG1 Fc region to construct a recombinant plasmid, which was transfected into HEK293E suspension cells for expression and purification to obtain the anti-PSMA Ab. Finally, the purity of the nanobody was evaluated by SDS-PAGE, and the binding affinity and specificity of the anti-PSMA Ab to the PSMA antigen were verified by ELISA and flow cytometry.

### Patient-derived organoid culture and lactate dehydrogenase assay

Tissue organoids were derived from the samples of a patient with PCa treated at Beijing Tsinghua Changgung Hospital and established by K2 Oncology Co., Ltd., according to a previous report (24). Briefly, fresh tumor tissues were cut into small pieces and cultured in a medium containing Matrigel to promote the formation of organoids from the tissue pieces. During the culture process, the growth of the organoids was observed regularly, and they were identified by morphological and molecular biological methods. This study was approved by the Ethics Committee of Beijing Tsinghua Changgung Hospital (ethical approval number: IRB24351-0-01). The PDO model was evaluated using hematoxylin and eosin (H&E) staining for histomorphological analysis, and PSMA IHC staining for characteristic confirmation. The cytotoxicity of NK cells against PDO, with or without the anti-PSMA Ab, was measured using a human LDH cytotoxicity assay kit (BioLegend), according to the manufacturer’s instructions. The OD of the wells was measured at 490 nm using an ELISA plate reader (BMG Omega), according to our previously reported method (25). The cytotoxicity was calculated as follows: (OD_sample_ − OD_low control_)/(OD_high control_ − OD_low control_) × 100%.

### *In vivo* anti-tumor activity study

Five-week-old male NOD-scid IL2rg−/− mice (NPGTM, VITALSTAR) were housed according to protocols approved by the Ethical Committee of Beijing Tsinghua Changgung Hospital (ethical approval number: IRB24351-0-01). We used a xenograft CRPC mouse model to evaluate the antitumor effects of combined treatment with NK cells and the constructed anti-PSMA Ab. The mice were subcutaneously injected with 22RV1 cells (2 × 10^6^ 22RV1 cells/mouse) on day zero and randomly divided into four groups (n = 6 each) on day 9 when bearing similar-sized tumors. The groups were labeled (1) control, (2) NK, (3) NK + IgG, and (4) NK + anti-PSMA Ab. Anti-PSMA Ab (10 mg/kg), IgG1 isotype (Med Chem Express, HY-P99001; 10 mg/kg), or PBS were administered intraperitoneally on days 10 and 18 after tumor inoculation. NK cells were administered by injecting 1 × 10^7^ NK cells via the tail vein on days 11, 15, 19, and 23, two of which were applied one day after anti-PSMA Ab or IgG therapy. Mice in the control group received the same volume of PBS as those in the treatment group. Tumor volumes were calculated using the following formula: Volume = L × W^2^/2, where L and W indicate the longest (L) and shortest (W) diameters of the implanted tumor, respectively, measured using a caliper. All mice were euthanized on day 28, and the implanted tumors were harvested, weighed, and collected for H&E staining. In addition, we established mouse satellite groups to monitor survival time (n = 6 each).

### Statistical analysis

For normally distributed data, one-way analysis of variance (ANOVA), followed by Tukey’s *post hoc* and Student’s t-test, was used for comparing multiple and two independent groups, respectively. For data that were not normally distributed, the Kruskal–Wallis and Mann–Whitney U tests were used for comparisons between multiple and two groups, respectively. Categorical variables were analyzed using the chi-square test. The Kaplan–Meier method was used to plot survival curves, and the log-rank test was used to determine the statistical significance of survival in *in vivo* experiments. Data analysis was conducted using SPSS (version 22.0; SPSS Inc., Chicago, IL, USA) and GraphPad Prism 8. Two-sided p < 0.05 was considered statistically significant. The schematic figure was drawn with FigDraw (www.figdraw.com).

## Results

### PSMA expression in PCa cell lines and tissues

**Figure 1:**
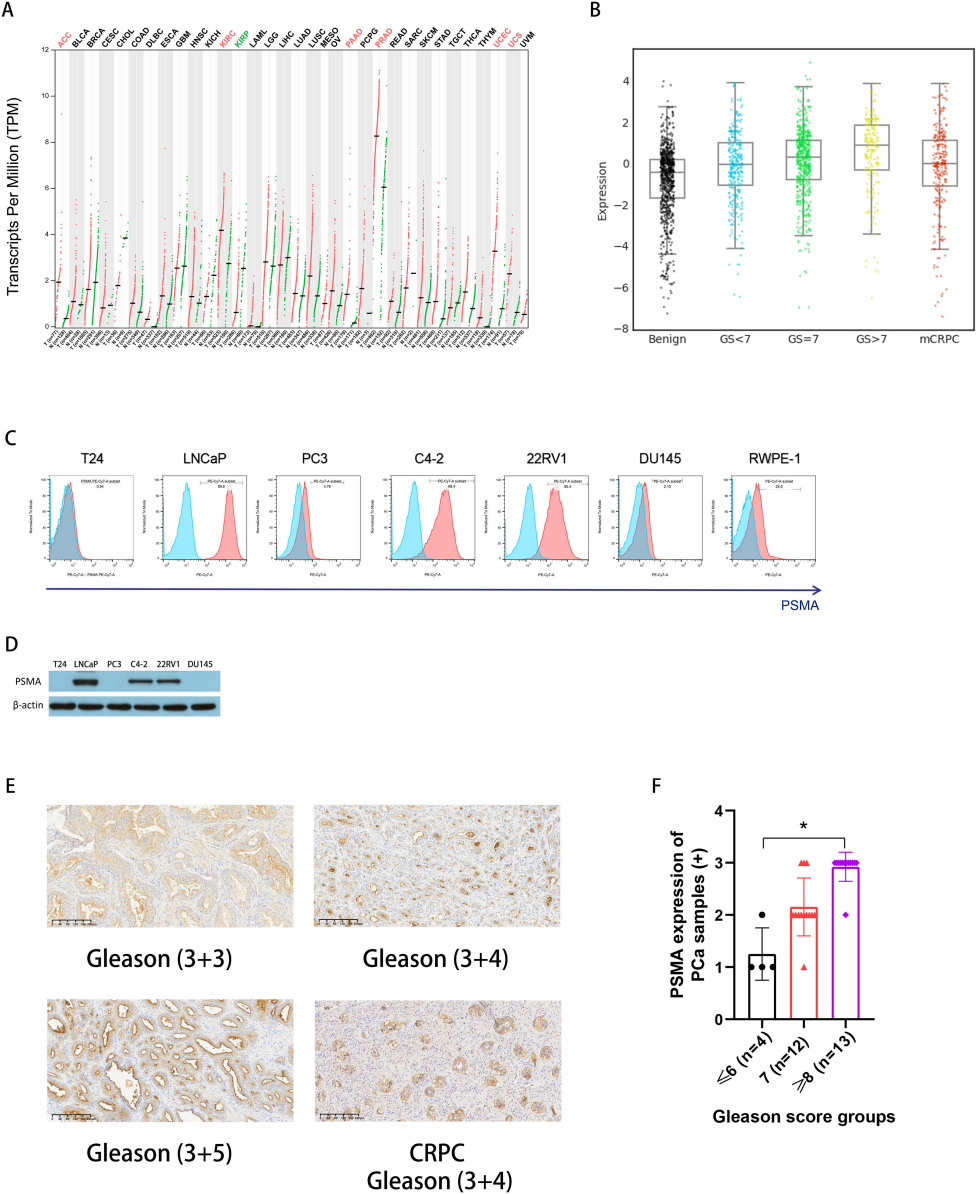
PSMA expression in PCa. **(A)** PSMA gene (FOLH1) expression was increased in PCa according to pan-cancer analysis using the GEPIA website (http://gepia.cancer-pku.cn/); **(B)** An analysis of the PCTA (http://www.thepcta.org/) revealed significant differences in PSMA gene expression among BPH, PCa with different grades, and CRPC; **(C)** PSMA expressions on bladder cancer cell line T24, PCa cell lines including LNCaP, PC3, C4-2, 22RV1, and DU145, and prostate epithelial cell line RWPE-1 were detected using flow cytometry; **(D)** Representative western blotting of PSMA protein levels in a panel of cancer cells, β-actin was used as a sample loading control; **(E)** Representative immunohistochemistry (IHC) images of PSMA from PCa specimens with different grades including one CRPC; **(F)** The summary data of the staining scores from PCa specimen with different grades: Gleason score ≤ 6 (n = 4), 7 (n = 12), ≥ 8 (n = 13), and the PSMA expression was compared among groups. Bars represent the means ± SD, one-way analysis of variance (ANOVA) followed by a Tukey *post hoc* test were used for multiple group comparisons. *p < 0.05. PSMA, prostate-specific membrane antigen; PCa, prostate cancer; GEPIA, Gene Expression Profiling Interactive Analysis; PCTA, Prostate Cancer Transcriptome Atlas website; BPH, benign prostatic hyperplasia; CRPC, castration-resistant prostate cancer;.

We examined PSMA expression in PCa cells using database, cell lines, and human PCa samples. Among the multiple types of cancers, FOLH1 (the gene encoding the PSMA protein) was highly expressed in prostate adenocarcinoma (PRAD), according to a pan-cancer analysis using the GEPIA website (**Figure 1A**). Furthermore, data from the PCTA website showed that the expression of FOLH1 mRNA in PCa tissue was higher than that in normal prostate tissue (p < 0.001) and was positively correlated with the Gleason score. Notably, FOLH1 expression was lower in mCRPC than in primary PCa (Gleason score ≥ 7 subgroup) (p = 0.028). (**Figure 1B**). As shown in **Figure 1C**, flow cytometry demonstrated that PSMA was highly expressed in PCa cell lines, including LNCaP (99.8%), C4-2 (88.4%), and 22RV1 (80.4%); moderately expressed in the normal prostate epithelial cell line RWPE-1 (25.0%); and lowly expressed in PCa cell lines PC3 (4.76%) and DU145 (2.10%) and BCa cell line T24 (0.54%). Consistent with the flow cytometry results, western blot analysis revealed similar PSMA expression levels in the cell lines (**Figure 1D**). Moreover, compared with normal prostate tissues, PCa tissue samples, including primary PCa cases and one CRPC case, displayed strong positive staining for PSMA (**Figure 1E**): 13 cases with a staining score of 3+ (Gleason score ≥ 8), 12 cases with a score of 2+ (Gleason score = 7), and 4 cases with a score of 1+ (Gleason score ≤ 6) (**Figure 1F**). Notably, PSMA expression in PCa specimens was positively correlated with the Gleason score. Collectively, our data shows that PSMA is a specific antigen that is highly expressed on the surface of PCa cells and could be a valid target for PCa immune cell treatment.

### Preparation of NK cells

**Figure 2:**
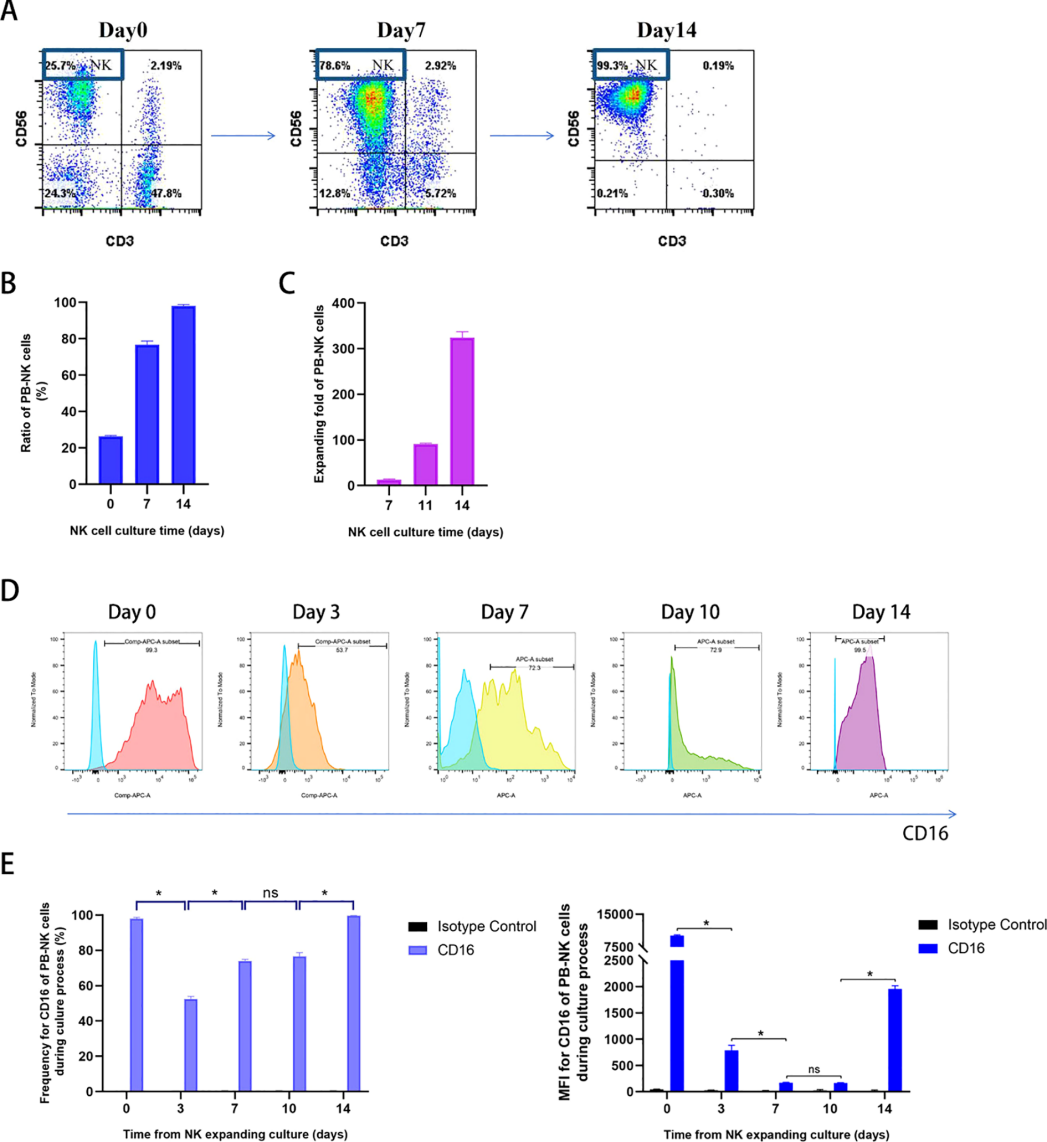
Identification of PB-NK cells cultured from healthy donors and measurement of CD16 expression on NK cells during the culture process. **(A)** Representative flow cytometry plots showing NK and T cell percentages. NK cells (CD3^−^CD56^+^) account for 25.7% in fresh PBMC; On days 7 and 14 after culture, the NK cells account for 78.6% and 99.3% in expanded fresh cells, respectively; **(B)** Summary data of PB-NK cell percentages on day 0, 7, 14 during NK cell culture (n = 3); **(C)** Summary data of PB-NK cell expanding fold (n = 3); **(D)** Representative flow cytometry plots of CD16 expression on CD3^−^CD56^+^ NK cells on day 0, 3, 7, 10, and 14 during PB-NK culture process; **(E)** Summary data of CD16 expression on CD3^−^CD56^+^ NK cells on day 0, 3, 7, 10, and 14 during PBNK culture process (n = 3). Data expressed as the means ± SD were plotted **(B, C, E)**, and ANOVA followed by a Tukey’s *post hoc* test were used for multiple group comparisons **(E)**. *p < 0.05; ns, not significant. PB-NK, peripheral blood-derived natural killer cell; PBMC, peripheral blood mononuclear cell.

As previously reported, we used an efficient manufacturing method to produce high-quality NK cells from the peripheral blood drawn from healthy donors over a period of 2 weeks (11). As shown in **Figure 2A**, NK cells (CD3^−^ CD56^+^) account for 25.7% of fresh PBMC in one healthy donor. During primary cell culture, NK cell purity gradually increased from 76.8 ± 3.5% on day 7 to 98.1 ± 1.2% on day 14 (**Figure 2B**), with little contamination by other immune cells. Moreover, NK cells stably expanded up to approximately 300-fold from the PBMCs after 2 weeks (**Figure 2C**). The expression of CD16 on NK cells during cell culture was monitored (**Figures 2D, E**), and although there were fluctuations, CD16 was abundantly expressed on the surface of mature NK cells when harvested.

### Isolation of anti-PSMA VHH clones with high affinity and specificity

**Figure 3:**
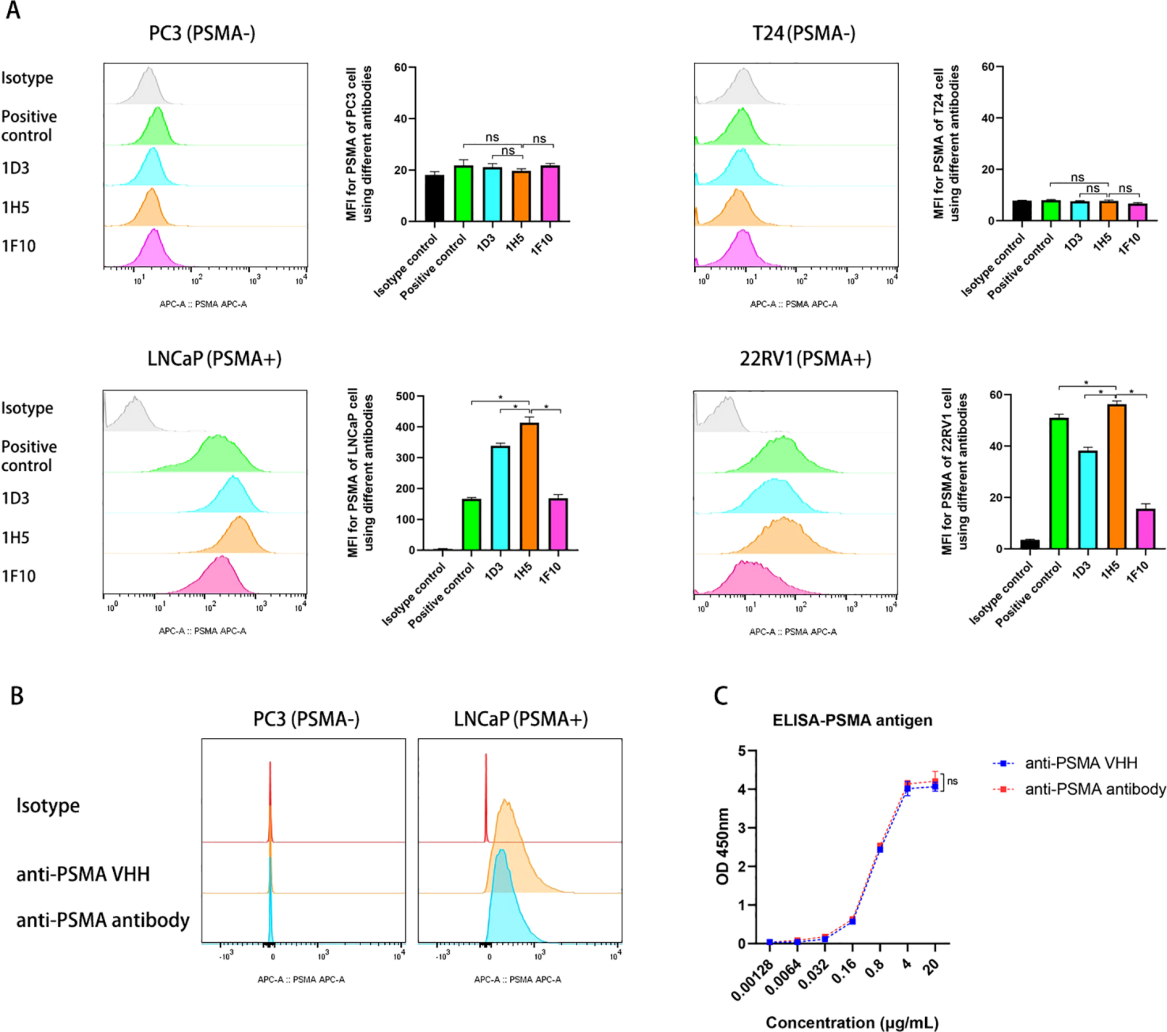
Evaluation of the selectivity of the VHHs and constructed anti-PSMA Ab toward PSMA antigens. **(A)** Evaluation of the binding of anti-PSMA VHHs (1D3, 1H5, and F10 clones) to PC3 cells (PSMA -), T24 cells (PSMA -), LNCaP cells (PSMA +), and 22RV1 cells (PSMA +) by flow cytometry. PSMA mAb (Biolegend, LNI-17 clone) against PSMA was used as a positive control; **(B)** Evaluation of the binding of anti-PSMA VHH (1H5 clone) and the constructed anti-PSMA Ab to PSMA negative PC3 and PSMA positive LNCaP prostate cancer cell lines; **(C)** Comparison of the anti-PSMA VHH with anti-PSMA Ab for binding to PSMA antigen by ELISA. Data expressed as means ± SD were plotted, and unpaired t-tests were used to compare two groups **(A, C)**. *p < 0.05; ns, not significant. VHH, variable domains of camelid heavy-chain antibody; anti-PSMA Ab, anti-prostate-specific membrane antigen antibody; ELISA, enzyme linked immunosorbent assay.

After 5 rounds of immunization, llama blood was drawn and PBMCs were isolated. This was followed by RNA extraction, cDNA synthesis, and phagemid library generation. After three rounds of phage display, 480 colonies were selected and lysed to obtain periplasmic extracts. Lysates with the highest binding affinity toward the PSMA antigen were selected and examined for selectivity by ELISA. Nine colonies with positive results were selected and sequenced, and nine VHH clones with known sequences were cloned into a plasmid vector to produce anti-PSMA VHHs (**Supplementary Material 1**). PSMA (+) cell lines, including LNCaP and 22RV1, and PSMA (−) cell lines, including PC3 and T24, were used to assess the specificity of the nine VHHs toward the PSMA antigen. Only three anti-PSMA VHHs (1D3, 1F10, and 1H5) with high binding affinity and specificity were selected (**Figure 3A**). Notably, the mean fluorescence intensity of 1H5 was significantly higher than that of the other VHHs when bound to PSMA-positive cells (p < 0.05). Therefore, we selected 1H5 anti-PSMA VHH because of its high specificity toward the PSMA antigen for further construction of the anti-PSMA Ab.

### Construction of anti-PSMA Ab

Using recombinant engineering, an anti-PSMA Ab was constructed by fusing the 1H5 anti-PSMA VHH with an Fc fragment (**Supplementary Material 2**). Recombinant genes were cloned into plasmids and used to produce antibodies in HEK293E suspension cells. The SDS-PAGE data showed that the purified Ab had a purity > 90%. Binding of the anti-PSMA Ab to the PSMA antigen was evaluated by ELISA and flow cytometry. These data showed that the affinity of the constructed anti-PSMA Ab for the PSMA antigen remained intact, and there was no significant difference in affinity and specificity between the anti-PSMA VHH and the newly constructed anti-PSMA (p > 0.05) (**Figures 3B, C**).

### The constructed anti-PSMA Ab enhanced NK cell cytotoxicity *in vitro*

**Figure 4:**
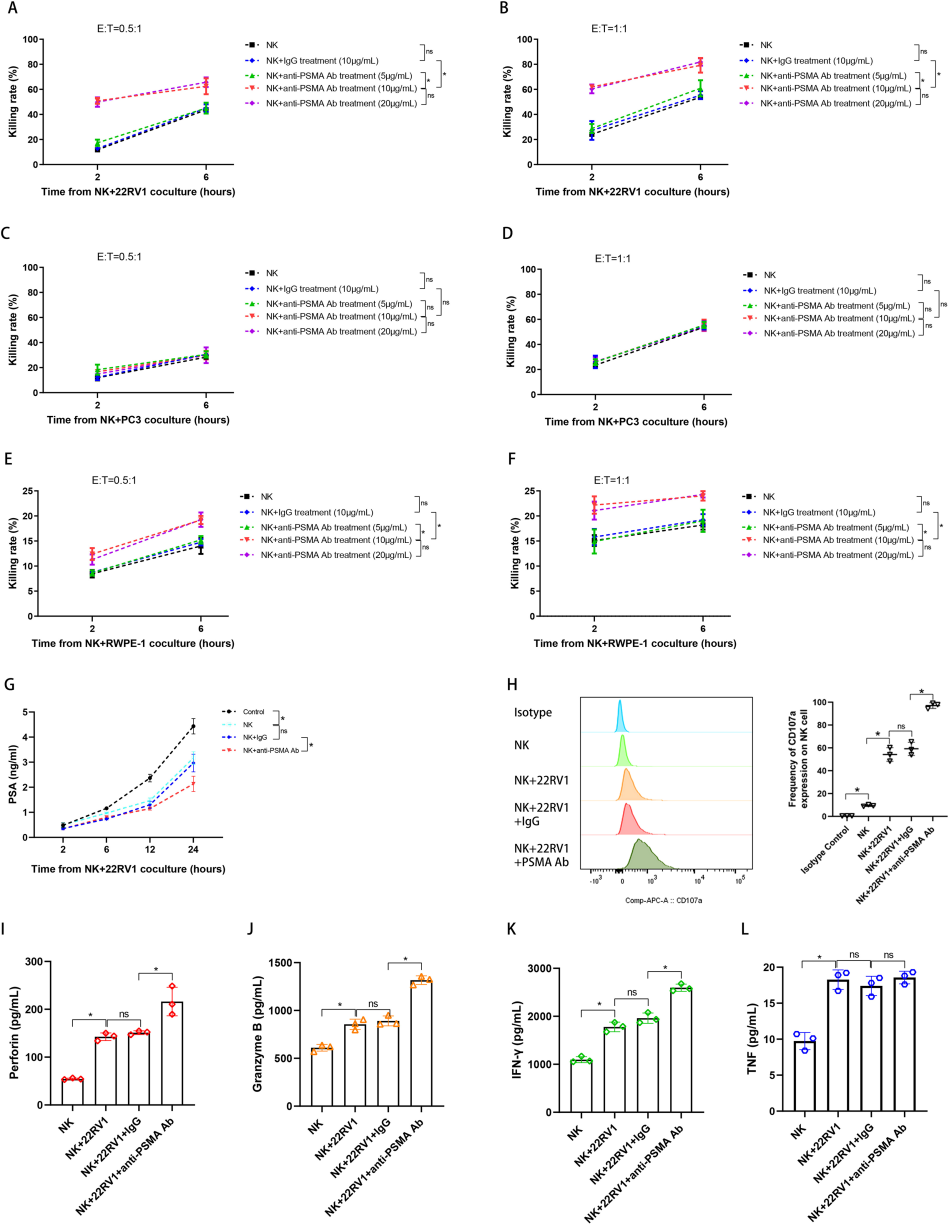
Evaluation of the cytotoxic activity of NK cells treated with anti-PSMA Ab *in vitro*. **(A, B)** The killing rates of NK cells against 22RV1 cells (PSMA strongly positive) in the NK, NK + IgG (10 μg/mL), NK + anti-PSMA Ab (5, 10, 20 μg/mL) treatment groups measured using the Cell Counting Kit-8 (CCK-8) assay at 2 and 6 h after co-culturing (n = 3). E/T = 0.5:1 **(A)**, 1:1 **(B)**, respectively; **(C, D)** The killing rates of NK cells against PC3 cells (PSMA negative) in the NK, NK + IgG (10 μg/mL), NK + anti-PSMA Ab (5, 10, 20 μg/mL) treatment groups measured using the CCK-8 assay at 2 and 6 h after co-culturing (n = 3). E/T = 0.5:1 **(C)**, 1:1 **(D)**, respectively; **(E, F)** The killing rates of NK cells against RWPE-1 cells (PSMA moderately positive) in the NK, NK + IgG (10 μg/mL), NK + anti-PSMA Ab (5, 10, 20 μg/mL) treatment groups measured using the CCK-8 assay at 2 and 6 h after co-culturing (n = 3). E/T = 0.5:1 **(E)**, 1:1 **(F)**, respectively; **(G)** PSA levels in the culture supernatant of NK cells co-cultured with 22RV1 cells in the control and treatment groups, including NK, NK + IgG, and NK + anti-PSMA Ab (10 μg/mL), measured via ELISA at 2, 6, 12, and 24 h after co-culturing (n = 3); **(H)** Representative flow cytometry plots and summary data (n = 3) of the MFI for CD107a expression in NK cells co-cultured with 22RV1 cells in the presence of anti-PSMA Ab (10 μg/mL) or IgG control (10 μg/mL). CD107a expression in NK cells was set as a negative control. Degranulation of NK cells was induced upon interaction with 22RV1 cells at a 1:1 ratio with anti-PSMA Ab or IgG for 6 h at 37 °C, the GolgiStop protein transport inhibitor was added during the final 2 h of the culture and NK cells were collected for cytometry measurement; **(I, J)** Comparison of supernatant perforin **(I)** and granzyme B **(J)** levels among NK cells co-cultured with 22RV1 cells and their counterparts co-cultured with 22RV1 cells in the presence of IgG control (10 μg/mL) or anti-PSMA Ab (10 μg/mL) at E:T of 1:1 after 6h coculture (n = 3). NK cells alone were set as negative control; **(K, L)** Comparison of IFN-γ **(K)** and TNF-α **(L)** levels among NK cells co-cultured with 22RV1 cells and their counterparts co-cultured with 22RV1 cells in the presence of IgG control (10 μg/mL) or anti-PSMA Ab (10 μg/mL) at E:T of 1:1 after 6h co-culture (n=3). NK cells alone were set as negative control; Data expressed as means ± SD were plotted, and ANOVA followed by a Tukey’s *post hoc* test was used to compare three or more groups **(A–L)**. *p < 0.05; ns, not significant. anti-PSMA Ab, anti-prostate-specific membrane antigen antibody; PSA, prostate-specific antigen; ELISA, enzyme-linked immunosorbent assay; IFN-γ, interferon-γ; TNF-α, tumor necrosis factor-α; E:T, effector-to-target ratio.

We performed CCK-8 assay and chose PSMA-negative PC3 cell line and a humanized IgG1 mAb as controls to evaluate whether the constructed anti-PSMA Ab could help NK cells target the PSMA molecule expressed on PCa cells, thus increasing NK cell cytotoxicity. Our CCK-8 assay data demonstrated that for the PSMA (+) cell line 22RV1, the anti-PSMA Ab at two concentrations enhanced the killing rate of NK cells at two different E/T ratios when compared to IgG or the absence of any antibody (**Figures 4A, B**). On the contrary, the anti-PSMA Ab, even at a high concentration of 20 μg/mL, did not promote NK cell cytotoxicity against the PSMA (−) cell line PC3 when compared with IgG (**Figures 4C, D**). Moreover, the low cytotoxicity of NK cells against the normal prostate epithelial cell line RWPE-1, which moderately expresses the PSMA protein, was increased by the anti-PSMA Ab at two concentrations (**Figures 4E, F**). Additionally, combined treatment with anti-PSMA Ab and NK cells delayed the elevation of supernatant PSA produced by 22RV1 cells compared to treatment with NK alone or NK + IgG (**Figure 4G**), which supported the CCK-8 assay results. Furthermore, activated NK cells expressed higher levels of CD107a, an NK cell degranulation marker, in the presence of an anti-PSMA Ab than in the absence of any antibody or in the presence of IgG after co-culturing with 22RV1 cells (E/T = 1:1, 6 h) (**Figure 4H**). To directly explore the cytotoxic proteins during degranulation and secreted cytokines, we measured the levels of perforin, granzyme B, IFN-γ, and TNF-α in the supernatant. Consistent with the CD107a results, the perforin and granzyme B levels from NK and 22RV1 cocultures in the presence of anti-PSMA Ab were higher than those in the absence of anti-PSMA Ab (**Figures 4I, J**). In addition, anti-PSMA Ab induced more release of IFN-γ from the activated NK cells that were incubated with 22RV1 for 6 hours (**Figure 4K**). However, we did not observe any changes in TNF-α release from the activated NK cells in the presence of anti-PSMA Ab (**Figure 4L**). In summary, these data suggest that anti-PSMA Abs can specifically stimulate NK cells to release more cytotoxic proteins and cytokines and possess more powerful anti-tumor effects against PSMA (+) PCa cells than non-PSMA-targeting Ab.

### The anti-PSMA Ab enhanced NK cell anti-tumor immunity in the PCa PDO model

**Figure 5:**
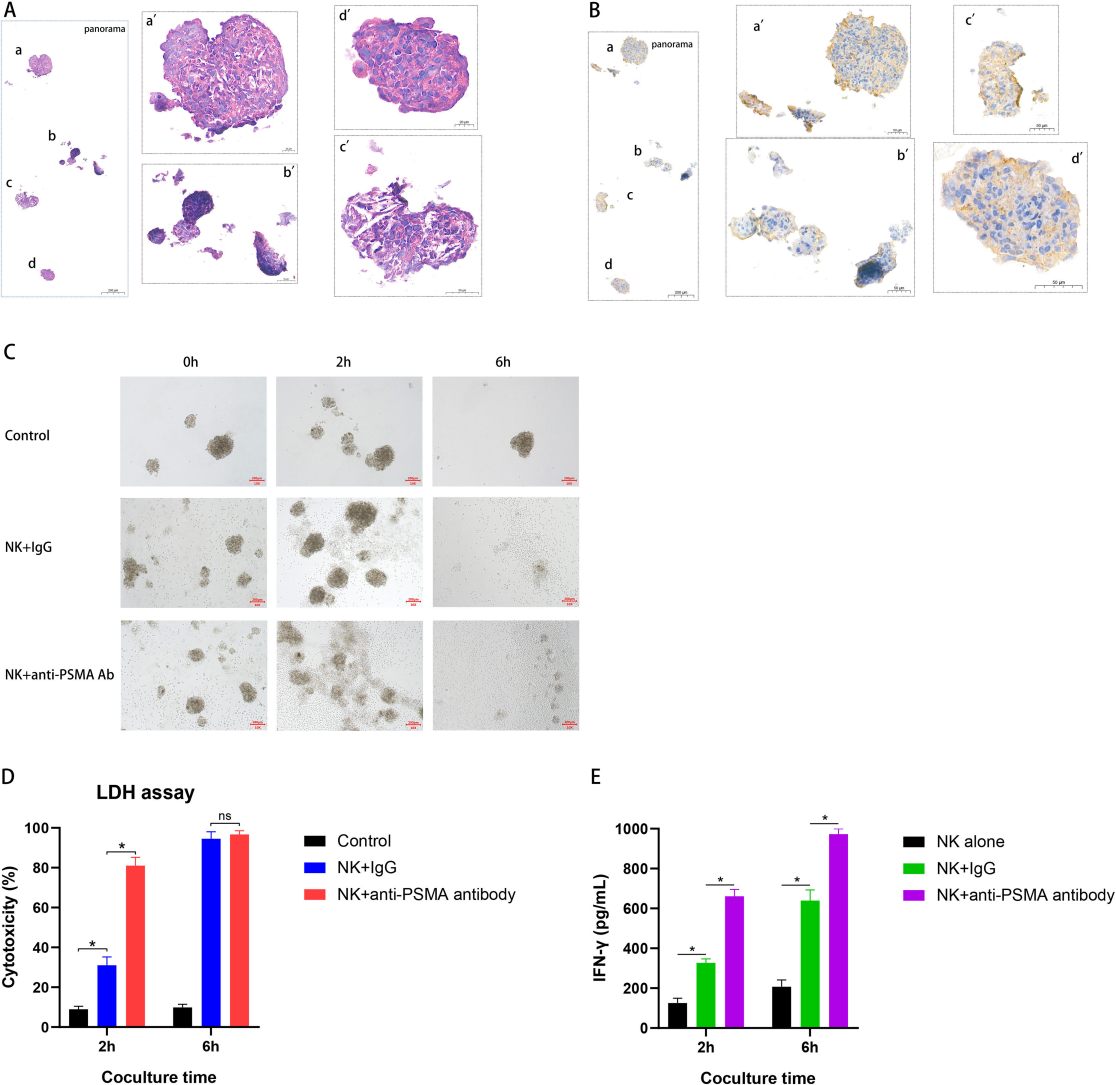
Development of PDO PCa models and cytotoxicity of combined treatment with anti-PSMA antibody and human peripheral blood-derived NK cells against the PDO. **(A)** Representative hematoxylin-and-eosin images of PCa tissue-derived organoid derived from PCa specimens (scale bar 200 μm). The left image was the panorama, and the right four images were local magnifications part by part (a→a’, b→b’, c→c’, d→d’); **(B)** Representative PSMA immunohistochemistry images of PCa tissue-derived organoid derived from PCa specimens (scale bar 200 μm). The left image was the panorama, and the right four images were local magnifications part by part (a→a’, b→b’, c→c’, d→d’); **(C)** Representative bright-field image of coculture of NK cells with PCa tissue-derived organoid in the presence of IgG or the constructed anti-PSMA antibody after 2 h and 6h coculture, PDOs alone were set as controls (n = 3); **(D)** Cytotoxicity of NK cells with IgG or anti-PSMA antibody (10 μg/mL) against PCa tissue-derived organoid at E/T ratio of 5:1 after 2 h and 6h coculture measured using LDH assay (n = 3); **(E)** IFN-γ levels of the supernatant after the NK cells were co-cultured with PCa tissue-derived organoid at E/T ratio of 5:1 after 2 h and 6h coculture measured using ELISA (n = 3). Data are shown as mean ± SD. Statistical significance was determined using an unpaired t-test **(D, E)**. *p < 0.05; ns, not significant. PDO, patient-derived organoid; PCa, prostate cancer; PSMA, prostate-specific membrane antigen; IgG, immunoglobulin G; E/T, effector-to-target ratio; LDH, lactate dehydrogenase; IFN-γ, interferon-gamma; ELISA, enzyme-linked immunosorbent assay.

As PDO models can accurately and efficiently recapitulate tissue architecture and function, we established a PCa PDO culture model using fresh tumor tissues from a patient with CRPC (for patient information, see **Supplementary Material 3**) and evaluated the anti-tumor effect of the combined treatment. H&E staining (**Figure 5A**) showed that the PDO model retained the pathological features of PCa tissue. PSMA examination by IHC demonstrated that PSMA was widely and highly expressed in the PDO model (**Figure 5B**). As observed in the bright-field image, although PCa tissue-derived PDO was completely disintegrated at 6 h after co-cultured with NK cells in the presence of IgG or anti-PSMA Ab when compared with the control group, anti-PSMA Ab treatment enhanced the anti-tumor effects of NK cells against PCa PDO after co-culture for 2 h (**Figure 5C**). The LDH assay results demonstrated that NK cell cytotoxicity against PCa PDO was significantly enhanced in the presence of the anti-PSMA Ab (**Figure 5D**), which was consistent with the bright-field imaging results. Moreover, anti-PSMA Ab induced NK cells to secrete more IFN-γ in the PCa PDO model (**Figure 5E**). Collectively, these results indicated that combined treatment with NK cells and anti-PSMA Ab displayed stronger cytotoxicity and higher cytokine secretion against PCa tissues directly derived from patients, further supporting the clinical applicability of this treatment strategy.

### The anti-PSMA Ab augmented the anti-tumor effect of NK cells on CRPC xenograft *in vivo*

**Figure 6:**
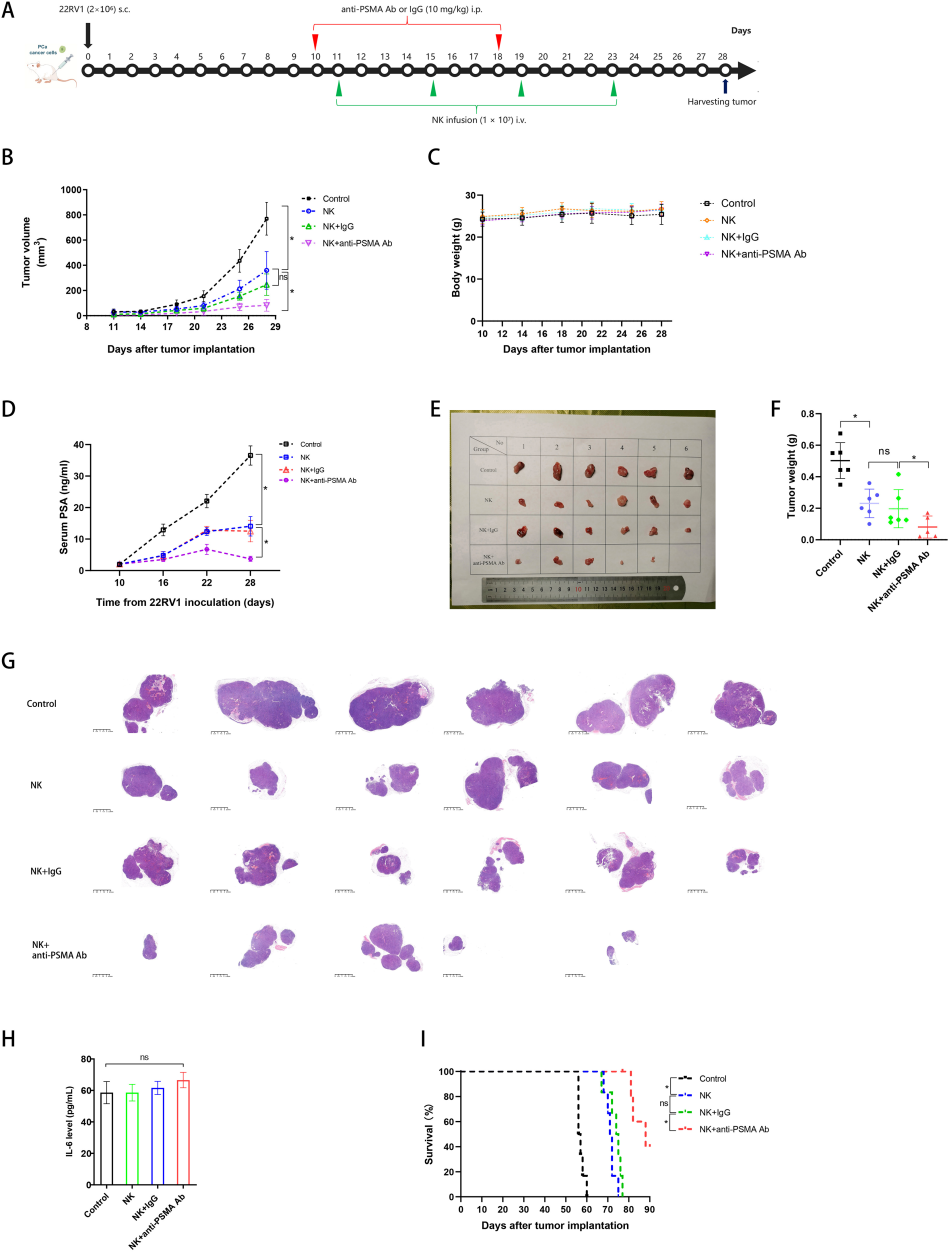
Anti-tumor effect of NK cells against CRPC in combination with anti-PSMA Ab in a subcutaneous tumor model *in vivo*. **(A)** Experimental protocol for the CRPC model used in **(B–I)**: mice were injected with PBS, anti-PSMA Ab or isotype-matched control mAb (10 mg/mg) intraperitoneally (i.p.) on days 10, 18 and injected with NK cells (1 × 10^7^) intravenously (i.v.) on days 11, 15, 19, and 23 after injections of 2 × 10^6^ 22RV1 cancer cells subcutaneously (s.c.) on day 0 (n = 6 per group); **(B)** Tumor volumes at various times (horizontal axis) after tumor inoculation in the control, NK, NK + IgG, and NK + anti-PSMA Ab groups. Tumor volumes were calculated according to the formula L × W^2^/2, where L and W represent the longest and shortest diameters measured using a caliper, respectively (n = 6 per group); **(C)** Body weights in the control and treatment groups over the whole treatment course (n = 6 per group); **(D)** Serum PSA levels of mice in the control, NK, NK + IgG, and anti-PSMA Ab groups (n = 6 per group) on days 10, 16, 22, and 28; **(E)** Images of tumors in mice 28 days after tumor inoculation (n = 5–6 in each group); **(F)** Tumor weights corresponding to each group when harvested on day 28 (n = 5–6 in each group); **(G)** HE examination of tumor specimen in the control and treatment groups on day 28 (n = 5–6 in each group); **(H)** Serum IL-6 levels in the control and treatment groups on day 28; **(I)** Cumulative Kaplan–Meier survival curves for mice (n = 6 per group) after tumor implantation. Data expressed as the means ± SD were plotted, and ANOVA followed by a Tukey’s *post hoc* test was used for multiple group comparisons **(B-D, F, H)**. The Kaplan–Meier method was used to estimate survival functions, and the log-rank test was used for group comparisons **(I)**. *p < 0.05; ns, not significant. CRPC, castration-resistant prostate cancer; PSMA, prostate-specific membrane antigen; Ab, antibody; PSA, prostate-specific antigen; IL-6, interleukin-6.

In the subcutaneous tumor model of CRPC (**Figure 6A**), the tumor sizes in the four groups were similar on day 9 before treatment (p > 0.05) (**Figure 6B**). NK cell treatment inhibited tumor growth compared to the control group, and anti-PSMA Ab therapy further augmented the tumor burden difference on days 14, 18, 21, 25, and 28 (**Figure 6B**).

The body weight of the mice did not differ among the four groups during the treatment process before day 28 (**Figure 6C**). Consistent with the tumor size findings, the elevation of serum PSA levels in the anti-PSMA Ab-combined NK cell therapy group was delayed compared to that in the control and NK cell monotherapy groups during the observation period between days 10 and 22; the PSA level in the combined therapy group started to dramatically decrease after day 22 (**Figure 6D**). After harvesting tumors from the mice on day 28, we photographed and weighed the tumors; the tumor masses in the NK cell + anti-PSMA Ab group were lower than those in the group treated with NK cells (no anti-PSMA Ab). It should be noted that no tumor burden was left in one inoculated mouse in the combined therapy group, reaching a “complete response” status (**Figures 6E, F**). From H&E staining of the maximal tumor specimen sections, we observed that the section area was positively correlated with tumor size and weight, and the tumors were separated into smaller lobes or cleared into 1–2 lobes left in the NK cell + anti-PSMA Ab group, compared with the other groups (**Figure 6G**). To evaluate the side effects of the combination therapy, we measured serum IL-6 levels and found that IL-6 levels in mice in the NK cell treatment groups were marginally higher than those in the control group (p > 0.05) (**Figure 6H**). The median survival times of the control, NK, NK + IgG, and NK + anti-PSMA Ab groups were 56, 71, 74, and 88 d, respectively. Mice in the NK + anti-PSMA Ab group survived longer than those in the other three groups (p < 0.05) (**Figure 6I**). Overall, the administration of the anti-PSMA Ab triggered more potent PSMA-dependent NK cell activation without severe toxic effects.

## Discussion

In this study, we immunized llamas with PSMA protein and LNCaP cell lysates to acquire anti-PSMA VHH with high affinity and specificity through screening and isolation. This VHH was then recombinantly fused with the human Fc region to produce an anti-PSMA Ab. We subsequently demonstrated that the constructed anti-PSMA Ab significantly enhanced specific antitumor effects against CRPC in adoptive human PB-NK cell therapy *in vitro*, in PDO, and in xenograft mouse models of PCa *in vivo*. This study is the first to explore the anti-tumor efficacy of combined anti-PSMA Ab and NK cell therapy against CRPC, the results of which could be rapidly translated into the clinic for patients with CRPC.

As the most common cancer in men, PCa ranks second in cancer-related mortality (1). Most patients with ADT-treated advanced PCa develop resistance and advance to CRPC, which usually results in poor survival with limited treatment options (2). Cancer immunotherapy, especially ICI, has gained traction in recent years owing to its notable success in treating multiple malignancies; however, this treatment strategy has shown a limited response in PCa due to multiple factors (3–6). Indeed, significant efforts have been made to improve PCa immunotherapies (26). We have previously demonstrated that high doses of NK cells exert antitumor effects against CRPC in a xenograft mouse model (11). To enable NK cells to recognize and kill PCa cells specifically and more efficiently and reduce the NK cell dosage, NK cells can be modified to harbor CARs, whereas BiKEs can be designed to combine NK cell therapies. In this study, we adopted the latter strategy to address the non-targeting obstacles in NK cell therapy. The specific Ab directs adoptive NK cells to tumors by simultaneously targeting one tumor-specific antigen on the PCa cell surface and one extracellular molecule CD16 on the NK cell surface.

It has been reported that PSMA is expressed at low levels in non-prostatic tissue and abundantly in the majority of PCa, with expression levels correlated with tumor stage and aggressiveness [ (19, 27), and see **Supplementary Material 4**]. In our study, we demonstrated that PSMA is highly expressed in multiple human PCa cell lines and tissue samples, including CRPC specimens from patients who have undergone RP, whereas it is only moderately expressed in normal prostate epithelial cell lines and lowly expressed in BCa cell lines. Additionally, our IHC results show that PSMA levels are positively correlated with PCa grade. Moreover, the results of bioinformatics analyses support our findings. Taken together, our results are consistent with the PSMA expression levels reported in the literature and suggest that PSMA could be an ideal target for the development of specific Ab and CRPC treatments. Several studies have confirmed that the Fc receptor CD16 (CD16A, FcγRIII) is the most effective activating receptor expressed in NK cells, resulting in ADCC-triggered tumor cell lysis when IgG-coated target cells bind to it and occur independently of other co-activating receptors (16, 17). To date, most adoptive NK cell therapies have involved the use of PB-NK cells, umbilical cord blood (UCB) NK cells, or NK-92 clonal cell lines; however, UCB NK cells and NK-92 cell lines lack the expression of CD16, while PB-NK cells highly express CD16 (16). In our study, we acquired high-quality NK cells with a purity of more than 95% from the peripheral blood of healthy donors after two weeks of expansion and validated that CD16 was abundantly expressed on the surface of mature NK cells. This is consistent with previous studies and has laid a solid foundation for combined therapy (16, 17).

Nanobodies are single-domain antibodies (VHH) devoid of light chains. They are found in camels and llamas and are the smallest antibody-based fragments displaying attractive features, such as being a stable construct, exhibiting greater hydrophilicity, displaying fast non-target tissue clearance and good tumor penetration capability, and displaying epitopes that are less antigenic for conventional antibodies (28). Here, we describe the generation and characterization of nanobodies directed against PSMA. Nine positive colonies were selected and sequenced after primary ELISA selection, and three of them (1D3, 1F10, and 1H5) were further selected according to their binding to naturally existing PSMA antigens using PSMA (+) and PSMA (−) cell lines. Finally, we chose 1H5 as the optimal VHH after PSMA-binding selection using flow cytometry to construct an anti-PSMA Ab. In addition, we demonstrated that the binding of our screened anti-PSMA VHH was no less than that of a commercial anti-PSMA Ab. It was reported that IgG1 demonstrates a high affinity for all FcγRs and is a potent activator of ADCC with less immunogenicity among all four IgG subclasses, including IgG1, IgG2, IgG3, and IgG4 (29). Therefore, in our study, we recombinantly fused VHH with the human IgG1 Fc region to produce an anti-PSMA Ab, and validated its affinity. Although the VHH is reformatted as an IgG by adding Fc fragment, the mentioned advantages could be partly preserved compared with conventional antibodies.

In our study, the anti-PSMA Ab significantly enhanced *in vitro* NK cell cytotoxicity against strongly PSMA (+) cells rather than the PSMA negative cells compared to that against NK cells or NK cells combined with IgG, as demonstrated by the CCK assay results. Interestingly, the anti-PSMA Ab also enhanced the cytotoxicity of NK cells when encountering normal prostate epithelial cells whose PSMA expression was moderate. The selective cytotoxicity of anti-PSMA Ab + NK cell therapy against cells with different levels of PSMA expression supports the clinical use of this combined therapy. The androgen-independent cell line 22RV1 was derived from a xenograft that was serially propagated in mice after castration-induced regression and relapse and produced PSA (30, 31), which is a routinely used biomarker for the diagnosis and prognosis of PCa in clinical settings. Consistent with the cytotoxicity results, the combination therapy significantly delayed PSA elevation compared with NK alone or NK + IgG therapy. Evidence indicates that early killing of NK cells mainly depends on granzyme B, whereas late cytotoxicity is mediated by death receptor (32). NK cell functional assay in our study showed that the anti-PSMA Ab upregulated CD107a expression in activated NK cells and induced NK cells to release more effector molecules, including perforin and granzyme B, 6 h after coculture, which supports our cytotoxicity results. Importantly, anti-PSMA Ab also increased the secretion of IFN-γ by NK cells. Production of IFN-γ by immune cells is generally antitumorigenic rather than pro-tumorigenic, which could promote the activation of inflammatory cells and their recruitment to the TME (33). Our results indicated that anti-PSMA Ab could help NK cells recruit more immune cells to make the TME hot. We did not observe an elevation of TNF-α, a strong proinflammatory cytokine, in the combined treatment group compared with NK alone or NK + IgG group, which could be a good sign of mild CRS.

As PDOs are a new biomedical research model that can reconstruct the phenotypic and genetic characteristics of the original tissue (34), we developed a PCa PDO model with high PSMA expression to validate the enhanced cytotoxicity of NK cells against PCa promoted by anti-PSMA Abs. The PDO results were consistent with our *in vitro* cell line data regarding cytotoxicity and cytokine secretion and provided further evidence for the clinical use of this combined therapy. Our *in vivo* xenograft study demonstrated that a four-dose regimen of PB-NK cells co-administered with two doses of anti-PSMA mAb mediated more potent activity and significantly improved survival compared with NK cell monotherapy or NK + IgG therapy. Notably, the inoculated tumor in one mouse was completely eliminated on day 28, and PSA levels started to decrease on day 22 in the NK + anti-PSMA mAb treatment group. The H&E staining results indicated that the PCa cells were separated into “islands” after NK cell-related treatments. In the NK + anti-PSMA mAb group, the tumor was either separated into multiple small “islands” or annihilated into 1–2 “islands,” or completely eliminated. We hypothesize that the infused NK cells infiltrated the tumor, divided it into several parts, and partially dissolved it. Compared to the control group, NK cell therapy did not influence body weight fluctuations or significantly increase serum IL-6 levels, a biomarker of CRS. Overall, the administration of NK cells with an anti-PSMA Ab significantly improved the therapeutic efficacy against CRPC *in vivo* compared to NK cell monotherapy without evident side effects, which is consistent with the *in vitro* results. NK cell-mediated ADCC is a key mechanism in killing cancer cells. Indeed, several therapeutic mAbs, such as rituximab (Rituxan^®^), cetuximab (Erbitux^®^), and trastuzumab (Herceptin^®^) based on this mechanism are in wide use and have shown therapeutic potential of NK cells (20). However, most preclinical and clinical trials have used bi- and trispecific NK cell engagers without adoptive NK cell infusion. For example, AFM13, a bispecific antibody targeting CD30 [a prominent marker of relapsed/refractory (R/R) Hodgkin lymphoma] and CD16A, mediates *in vivo* ADCC and NK cell retention activity (35, 36). We reviewed the related literature and found only one clinical trial that combined AFM13 and UCB-derived NK cells to treat R/R Hodgkin or non-Hodgkin lymphoma (NCT04074746) (20). In addition, only one preclinical study has explored the antitumor effect of combined anti-PSMA Ab with immune cells (37). This study showed that mouse–human chimeric IgG1 of PSMA-recognizing mouse mAb showed ADCC against PSMA-expressing PCa cells in the presence of human PBMCs rather than NK cells *in vitro* (37), which is consistent with our *in vitro* results. However, this study did not investigate the anti-tumor effect of the anti-PSMA Ab in the presence of human immune cells *in vivo*. In this study, immunocompromised mice lacking immune cells were used. As previously reported, activated adoptive NK cells secrete an array of chemokines to recruit other immune cells, including T cells, dendritic cells, macrophages, and NK cells, to the tumor site (25). In addition, the anti-PSMA Ab binding to the PCa cell surface can engage macrophage FcγRs to elicit ADCC. Therefore, the therapeutic efficacy of combined treatment against CRPC in clinical settings would be better than the positive results obtained in immunocompromised mice.

Our study has two main limitations. First, the human TME could hardly be simulated in our PCa xenograft model using immunocompromised mice, and future studies using humanized mouse models are required to validate our results. Second, we did not engineer the Fc region and directly fused the human IgG1 Fc region with VHH to construct an anti-PSMA Ab. Thus, the IgG Fc region of our anti-PSMA Ab may be molecularly engineered to further increase its affinity for the IgG Fc receptor CD16a to modulate effector functions.

## Conclusions

**Figure 7:**
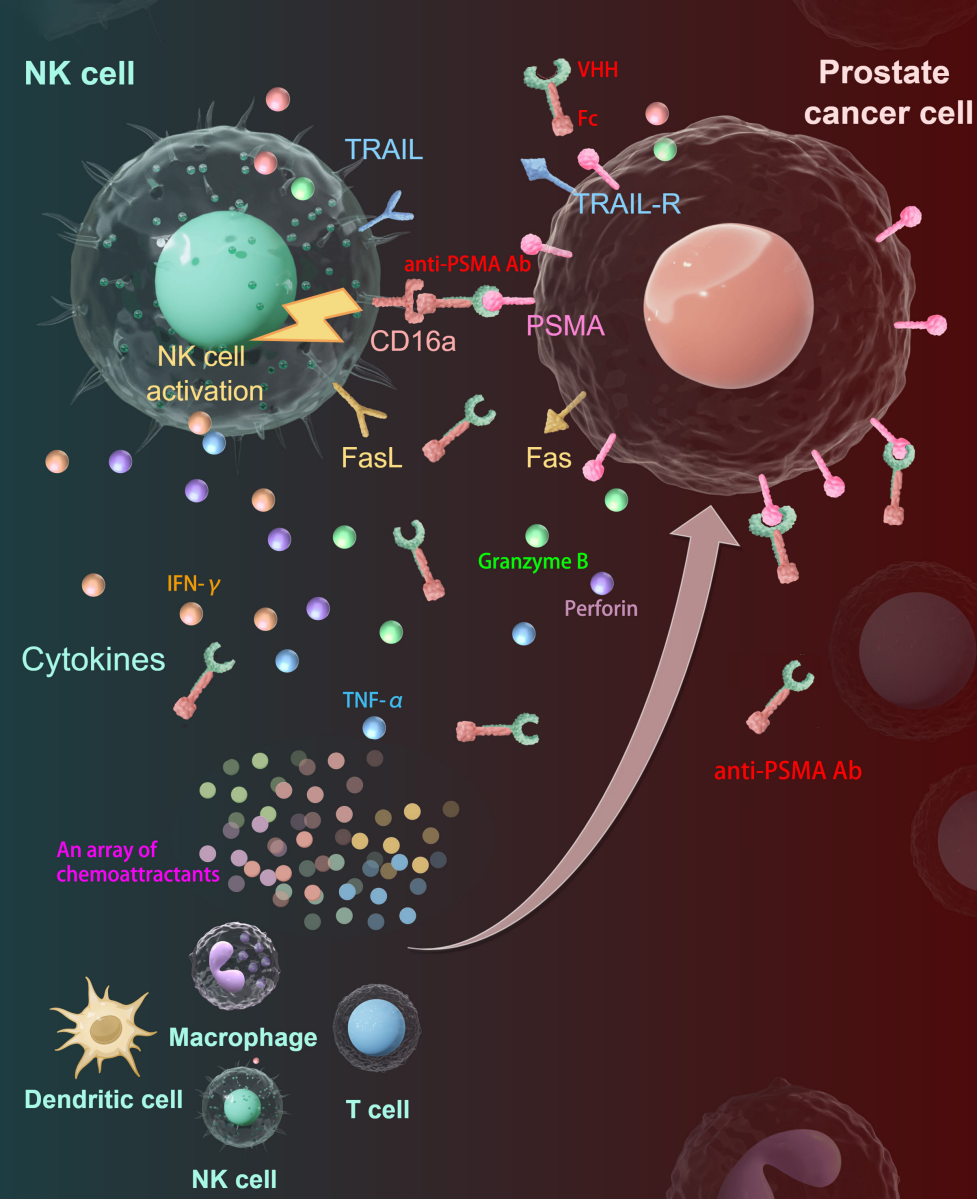
Summary diagram of our results. The anti-PSMA Ab was constructed by fusion of VHH and Fc fragments. The anti-PSMA Ab could direct NK cells to target PCa cells through the tumor-specific target molecule PSMA on one hand and to CD16-positive NK cells on the other hand, forming cytolytic synapses and activating NK cells to release cytotoxic granules containing perforin and granzymes, upregulate TRAIL and Fas-L to kill PCa cells. Besides, the activated NK cells could secret cytokines including IFN-γ and TNF-α to reshape the immune microenvironment. Moreover, the activated NK cells produce an array of chemokines to recruit other immune cells including T cells, macrophages, and dendritic cells to the tumor site, making the TME “hotter”. PSMA, prostate-specific membrane antigen; Ab, antibody; PCa, prostate cancer; TRAIL, tumor necrosis factor-related apoptosis-inducing ligand; TRAIL-R, TRAIL receptor; VHH, variable domain of heavy-chain-only antibody; IFN-γ, interferon-γ; TNF-α, tumor necrosis factor-α.

In summary, we successfully constructed a novel anti-PSMA Ab with high affinity and specificity toward the PSMA antigen and demonstrated that combined treatment with this anti-PSMA Ab and human PB-NK cells manufactured in our established NK cell expanding system enhanced the anti-tumor effect against CRPC *in vitro* and *in vivo*, which is a promising immunotherapeutic strategy for treating CRPC in clinical settings. As shown in **Figure 7**, the anti-PSMA Ab specifically directs NK cells to PCa sites by simultaneously binding to the specific antigen PSMA on the PCa cell surface and CD16 on the NK cell surface, activating NK cells to display potent anti-tumor activity. Activated NK cells secrete multiple chemokines to recruit other immune cells to the tumor site to convert a “cold” TME into a “hot” TME.

## Data Availability

The datasets presented in this study can be found in online repositories. The names of the repository/repositories and accession number(s) can be found in the article/**Supplementary Material**.

## References

[1] James ND Tannock I N’Dow J Feng F Gillessen S Ali SA. The Lancet Commission on prostate cancer: planning for the surge in cases. Lancet. (2024) 403:1683–722. doi: 10.1016/S0140-6736(24)00651-2 PMC7617369 38583453

[2] Wang F Li Z Feng X Yang D Lin M. Advances in PSMA-targeted therapy for prostate cancer. Prostate Cancer Prostatic Dis. (2022) 25:11–26. doi: 10.1038/s41391-021-00394-5 34050265

[3] Kantoff PW Higano CS Shore ND Berger ER Small EJ Penson DF. Sipuleucel-T immunotherapy for castration-resistant prostate cancer. N Engl J Med. (2010) 363:411–22. doi: 10.1056/NEJMoa1001294 20818862

[4] Kaye DR Lee HJ Gordee A George DJ Ubel PA Scales CD. Medication payments by insurers and patients for the treatment of metastatic castrate-resistant prostate cancer. JCO Oncol Pract. (2023) 19:e600–17. doi: 10.1200/OP.22.00645 PMC10113111 36689695

[5] Rekoske BT McNeel DG. Immunotherapy for prostate cancer: False promises or true hope? Cancer. (2016) 122:3598–607. doi: 10.1002/cncr.30250 PMC5115970 27649312

[6] Galustian C Dalgleish A Bodman-Smith M Kusmartsev S Dasgupta P. Editorial: Immunotherapy for Prostate Cancer-turning the immunological desert into an oasis of hope. Front Oncol. (2022) 12:1021870. doi: 10.3389/fonc.2022.1021870 36158670 PMC9495446

[7] Miller JS Soignier Y Panoskaltsis-Mortari A McNearney SA Yun GH Fautsch SK. Successful adoptive transfer and *in vivo* expansion of human haploidentical NK cells in patients with cancer. Blood. (2005) 105:3051–7. doi: 10.1182/blood-2004-07-2974 15632206

[8] Curti A Ruggeri L D'Addio A Bontadini A Dan E Motta MR. Successful transfer of alloreactive haploidentical KIR ligand-mismatched natural killer cells after infusion in elderly high risk acute myeloid leukemia patients. Blood. (2011) 118(12):3273–9. doi: 10.1182/blood-2011-01-329508 21791425

[9] Romee R Rosario M Berrien-Elliott MM Wagner JA Jewell BA Schappe T. Cytokine-induced memory-like natural killer cells exhibit enhanced responses against myeloid leukemia. Sci Transl Med. (2016) 8:357ra123. doi: 10.1126/scitranslmed.aaf2341 PMC5436500 27655849

[10] Handgretinger R Lang P André MC. Exploitation of natural killer cells for the treatment of acute leukemia. Blood. (2016) 127:3341–9. doi: 10.1182/blood-2015-12-629055 27207791

[11] Wang F Dong X Wang J Yang F Liu D Ma J. Allogeneic expanded human peripheral NK cells control prostate cancer growth in a preclinical mouse model of castration-resistant prostate cancer. J Immunol Res. (2022) 2022:1786395. doi: 10.1155/2022/1786395 35450395 PMC9017519

[12] Wang F Liu S Liu F Xu T Ma J Liang J. TIGIT immune checkpoint blockade enhances immunity of human peripheral blood NK cells against castration-resistant prostate cancer. Cancer Lett. (2023) 568:216300. doi: 10.1016/j.canlet.2023.216300 37414394

[13] Wang F Wu L Yin L Shi H Gu Y Xing N. Combined treatment with anti-PSMA CAR NK-92 cell and anti-PD-L1 monoclonal Ab enhances the antitumour efficacy against castration-resistant prostate cancer. Clin Transl Med. (2022) 12:e901. doi: 10.1002/ctm2.901 35696531 PMC9191826

[14] Hodgins JJ Khan ST Park MM Auer RC Ardolino M. Killers 2.0: NK cell therapies at the forefront of cancer control. J Clin Invest. (2019) 129:3499–510. doi: 10.1172/JCI129338 PMC6715409 31478911

[15] Chen Y Lu D Churov A Fu R. Research progress on NK cell receptors and their signaling pathways. Mediators Inflamm. (2020) 2020:6437057. doi: 10.1155/2020/6437057 32774149 PMC7396059

[16] Myers JA Miller JS. Exploring the NK cell platform for cancer immunotherapy. Nat Rev Clin Oncol. (2021) 18:85–100. doi: 10.1038/s41571-020-0426-7 32934330 PMC8316981

[17] Bryceson YT March ME Ljunggren HG Long EO. Activation, coactivation, and costimulation of resting human natural killer cells. Immunol Rev. (2006) 214:73–91. doi: 10.1111/j.1600-065X.2006.00457.x 17100877 PMC3845883

[18] Meng F Zhang S Xie J Zhou Y Wu Q Lu B. Leveraging CD16 fusion receptors to remodel the immune response for enhancing anti-tumor immunotherapy in iPSC-derived NK cells. J Hematol Oncol. (2023) 16:62. doi: 10.1186/s13045-023-01455-z 37316891 PMC10265820

[19] Wang Z Zhu B Jiang F Chen X Wang G Ding N. Design, synthesis and evaluation of novel prostate-specific membrane antigen-targeted aryl [18F]fluorosulfate PET tracers. Bioorg Med Chem. (2024) 106:117753. doi: 10.1016/j.bmc.2024.117753 38749342

[20] Huan T Guan B Li H Tu X Zhang C Tang B. Principles and current clinical landscape of NK cell engaging bispecific Ab against cancer. Hum Vaccin Immunother. (2023) 19:2256904. doi: 10.1080/21645515.2023.2256904 37772505 PMC10543353

[21] Evazalipour M Tehrani BS Abolhassani M Morovvati H Omidfar K. Camel heavy chain antibodies against prostate-specific membrane antigen. Hybridoma (Larchmt). (2012) 31:424–9. doi: 10.1089/hyb.2012.0048 23244321

[22] Behar G Sibéril S Groulet A Chames P Pugnière M Boix C. Isolation and characterization of anti-FcgammaRIII (CD16) llama single-domain antibodies that activate natural killer cells. Protein Eng Des Sel. (2008) 21:1–10. doi: 10.1093/protein/gzm064 18073223

[23] Broisat A Hernot S Toczek J De Vos J Riou LM Martin S. Nanobodies targeting mouse/human VCAM1 for the nuclear imaging of atherosclerotic lesions. Circ Res. (2012) 110:927–37. doi: 10.1161/CIRCRESAHA.112.265140 PMC3918224 22461363

[24] Zhang Q Deng T Zhang H Zuo D Zhu Q Bai M. Adipocyte-derived exosomal MTTP suppresses ferroptosis and promotes Chemoresistance in Colorectal Cancer. Adv Sci (Weinh). (2022) 9:e2203357. doi: 10.1002/advs.202203357 35978266 PMC9534973

[25] Wang F Zhang G Xu T Ma J Wang J Liu S. High and selective cytotoxicity of ex vivo expanded allogeneic human natural killer cells from peripheral blood against bladder cancer: implications for natural killer cell instillation after transurethral resection of bladder tumor. J Exp Clin Cancer Res. (2024) 43:24. doi: 10.1186/s13046-024-02955-7 38245792 PMC10799482

[26] Sridaran D Bradshaw E DeSelm C Pachynski R Mahajan K Mahajan NP. Prostate cancer immunotherapy: Improving clinical outcomes with a multi-pronged approach. Cell Rep Med. (2023) 4:101199. doi: 10.1016/j.xcrm.2023.101199 37738978 PMC10591038

[27] Dang K Castello G Clarke SC Li Y Balasubramani A Boudreau A. Attenuating CD3 affinity in a PSMAxCD3 bispecific antibody enables killing of prostate tumor cells with reduced cytokine release. J Immunother Cancer. (2021) 9:e002488. doi: 10.1136/jitc-2021-002488 34088740 PMC8183203

[28] Cortez-Retamozo V Lauwereys M Hassanzadeh Gh G Gobert M Conrath K Muyldermans S. Efficient tumor targeting by single-domain antibody fragments of camels. Int J Cancer. (2002) 98:456–62. doi: 10.1002/ijc.10212 11920600

[29] Yu J Song Y Tian W. How to select IgG subclasses in developing anti-tumor therapeutic antibodies. J Hematol Oncol. (2020) 13:45. doi: 10.1186/s13045-020-00876-4 32370812 PMC7201658

[30] Sramkoski RM Pretlow TG 2nd Giaconia JM Pretlow TP Schwartz S Sy MS. A new human prostate carcinoma cell line, 22Rv1. In Vitro Cell Dev Biol Anim. (1999) 35:403–9. doi: 10.1007/s11626-999-0115-4 10462204

[31] Paju A Hotakainen K Cao Y Laurila T Gadaleanu V Hemminki A. Increased expression of tumor-associated trypsin inhibitor, TATI, in prostate cancer and in androgen-independent 22Rv1 cells. Eur Urol. (2007) 52:1670–9. doi: 10.1016/j.eururo.2007.01.096 17306443

[32] Prager I Liesche C van Ooijen H Urlaub D Verron Q Sandström N. NK cells switch from granzyme B to death receptor-mediated cytotoxicity during serial killing. J Exp Med. (2019) 216:2113–27. doi: 10.1084/jem.20181454 PMC6719417 31270246

[33] Gocher AM Workman CJ Vignali DAA. Interferon-γ: teammate or opponent in the tumour microenvironment? Nat Rev Immunol. (2022) 22:158–72. doi: 10.1038/s41577-021-00566-3 PMC8688586 34155388

[34] Yang R Yu Y. Patient-derived organoids in translational oncology and drug screening. Cancer Lett. (2023) 562:216180. doi: 10.1016/j.canlet.2023.216180 37061121

[35] Rothe A Sasse S Topp MS Eichenauer DA Hummel H Reiners KS. A phase 1 study of the bispecific anti-CD30/CD16A antibody construct AFM13 in patients with relapsed or refractory Hodgkin lymphoma. Blood. (2015) 125:4024–31. doi: 10.1182/blood-2014-12-614636 PMC4528081 25887777

[36] Bartlett NL Herrera AF Domingo-Domenech E Mehta A Forero-Torres A Garcia-Sanz R. A phase 1b study of AFM13 in combination with pembrolizumab in patients with relapsed or refractory Hodgkin lymphoma. Blood. (2020) 136:2401–9. doi: 10.1182/blood.2019004701 PMC7685206 32730586

[37] Sugimoto Y Hirota M Yoshikawa K Sumitomo M Nakamura K Ueda R. The therapeutic potential of a novel PSMA antibody and its IL-2 conjugate in prostate cancer. Anticancer Res. (2014) 34:89–97. 24403448

