# Supplementary material for: Combined treatment with anti-PSMA antibody and human peripheral blood-derived NK cells for castration-resistant prostate cancer
Source: Front Immunol. 2025 May 21;16:1572676. doi: 10.3389/fimmu.2025.1572676 (PMC12133763; doi:10.3389/fimmu.2025.1572676)
Supplement: Supplementary file 2 [file DataSheet2.docx]

**Supplementary Material 2**

**The Construction and Panning of Alpaca Phage Immunological Library**

**Methods**

According to the codon preference of mammals, the target gene was artificially synthesized and inserted into the expression vector. The sequence of the target protein is as follows:

Amino acid sequence:

SNEATNITPKHNMKAFLDELKAENIKKFLYNFTQIPHLAGTEQNFQLAKQIQSQWKEFGLDSVELAHYDVLLSYPNKTHPNYISIINEDGNEIFNTSLFEPPPPGYENVSDIVPPFSAFSPQGMPEGDLVYVNYARTEDFFKLERDMKINCSGKIVIARYGKVFRGNKVKNAQLAGAKGVILYSDPADYFAPGVKSYPDGWNLPGGGVQRGNILNLNGAGDPLTPGYPANEYAYRRGIAEAVGLPSIPVHPIGYYDAQKLLEKMGGSAPPDSSWRGSLKVPYNVGPGFTGNFSTQKVKMHIHSTNEVTRIYNVIGTLRGAVEPDRYVILGGHRDSWVFGGIDPQSGAAVVHEIVRSFGTLKKEGWRPRRTILFASWDAEEFGLLGSTEWAEENSRLLQERGVAYINADSSIEGNYTLRVDCTPLMYSLVHNLTKELKSPDEGFEGKSLYESWTKKSPSPEFSGMPRISKLGSGNDFEVFFQRLGIASGRARYTKNWETNKFSGYPLYHSVYETYELVEKFYDPMFKYHLTVAQVRGGMVFELANSIVLPFDCRDYAVVLRKYADKIYSISMKHPQEMKTYSVSFDSLFSAVKNFTEIASKFSERLQDFDKSNPIVLRMMNDQLMFLERAFIDPLGLPDRPFYRHVIYAPSSHNKYAGESFPGIYDALFDIESKVDPSKAWGEVKRQIYVAAFTVQAAAETLSEVA

Gene sequence optimized according to the codon preference of mammalian cells:

AGCAACGAGGCCACCAACATCACCCCCAAGCACAACATGAAGGCCTTCCTGGACGAGCTGAAGGCCGAGAACATCAAGAAGTTCCTGTACAACTTCACCCAGATCCCTCACCTGGCCGGAACAGAGCAGAACTTCCAGCTGGCCAAGCAGATCCAGAGCCAGTGGAAGGAGTTCGGCCTGGATAGCGTGGAGCTGGCTCATTACGACGTGCTGCTGAGCTACCCCAACAAGACCCACCCCAACTACATCAGCATCATCAACGAGGACGGCAACGAGATCTTCAACACCAGCCTGTTTGAGCCTCCTCCTCCAGGATACGAGAACGTCTCCGACATCGTGCCCCCTTTCAGCGCCTTTAGCCCTCAGGGCATGCCAGAGGGAGACCTGGTCTACGTGAACTACGCCAGGACCGAGGACTTCTTCAAGCTGGAGCGGGACATGAAGATCAACTGCAGCGGCAAGATCGTGATCGCCAGATACGGCAAGGTGTTCCGGGGCAACAAGGTCAAGAACGCCCAGCTGGCAGGAGCCAAAGGCGTGATCCTGTACAGCGACCCAGCCGATTACTTTGCCCCAGGCGTGAAGAGCTACCCCGACGGTTGGAATCTGCCAGGAGGAGGCGTGCAGAGAGGCAACATCCTGAACCTGAATGGCGCCGGAGATCCTCTGACACCAGGATACCCAGCCAACGAGTACGCCTACAGGAGAGGAATCGCCGAAGCAGTGGGACTGCCTAGCATCCCCGTGCACCCTATCGGCTACTACGACGCCCAGAAGCTGCTGGAGAAGATGGGAGGCAGCGCTCCTCCAGATAGCTCTTGGAGAGGCAGCCTGAAGGTGCCATACAACGTGGGACCAGGCTTTACCGGCAACTTCAGCACCCAGAAGGTCAAGATGCACATCCACAGCACCAACGAGGTCACCCGGATCTACAACGTGATCGGCACACTGAGAGGAGCCGTGGAGCCAGACAGATACGTGATCCTGGGCGGCCACAGAGACTCTTGGGTGTTTGGCGGCATCGATCCTCAGAGCGGAGCAGCAGTGGTGCACGAGATCGTGAGAAGCTTCGGCACCCTGAAGAAGGAGGGTTGGAGGCCTAGGAGAACCATCCTGTTCGCCAGCTGGGACGCCGAAGAGTTTGGACTGCTGGGAAGCACCGAGTGGGCCGAAGAGAACAGCAGACTGCTGCAGGAGAGGGGAGTGGCTTACATCAACGCCGACAGCAGCATCGAGGGCAACTACACACTGCGCGTGGATTGCACCCCTCTGATGTACAGCCTGGTGCACAACCTGACCAAGGAGCTGAAGAGCCCCGACGAAGGATTTGAGGGCAAGAGCCTGTACGAGTCTTGGACCAAGAAGAGCCCTTCTCCCGAGTTTAGCGGCATGCCTAGGATCAGCAAGCTGGGAAGCGGCAACGACTTCGAGGTGTTCTTCCAGCGGCTGGGCATTGCCAGCGGCAGAGCCAGGTACACCAAGAATTGGGAGACCAACAAGTTCAGCGGCTACCCCCTGTACCACAGCGTGTACGAGACCTACGAGCTGGTGGAGAAGTTCTACGACCCCATGTTCAAGTACCACCTGACCGTGGCTCAGGTCCGAGGAGGAATGGTGTTCGAGCTGGCCAACAGCATCGTGCTGCCCTTCGATTGCCGGGACTACGCAGTGGTGCTGAGGAAGTACGCCGACAAGATCTACAGCATCAGCATGAAGCACCCCCAGGAGATGAAGACCTACAGCGTGTCCTTCGACAGCCTGTTCAGCGCCGTGAAGAACTTCACCGAGATCGCCAGCAAGTTCAGCGAGAGGCTGCAGGACTTCGACAAGAGCAACCCCATCGTGCTGCGGATGATGAACGACCAGCTGATGTTCCTGGAGAGGGCCTTCATCGATCCTCTGGGACTGCCAGACAGACCCTTCTACAGGCACGTGATCTACGCCCCTAGCAGCCACAACAAGTACGCCGGAGAGAGCTTTCCAGGCATCTACGACGCCCTGTTCGACATCGAGAGCAAGGTGGACCCTAGCAAGGCTTGGGGCGAAGTGAAGAGGCAGATCTACGTGGCCGCCTTCACAGTGCAGGCAGCCGCCGAAACACTGTCTGAAGTGGCT

Insert it into the self-developed vector. The structure of the vector is relatively close to the following vectors:


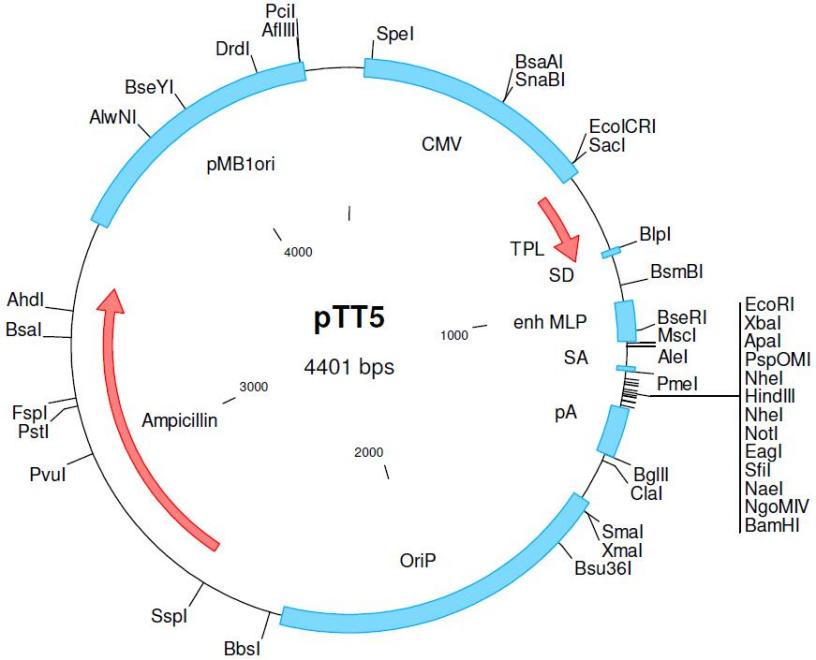


Identification of positive clones: Verified by sequencing, the synthesized sequence was correct.

Small-scale expression verification

1.Transform the constructed plasmid into TOP10B competent cells, conduct large-scale cultivation, and prepare the plasmid using a plasmid large extraction kit.

2.Cultivate 293f cells in large quantities in a 37°C CO₂ constant temperature incubator shaker with 5% CO₂ at 120 rpm.

3.One day before transfection, collect the cells by centrifuging at 1000 rpm for 5 minutes, resuspend them in 293f expression medium, adjust the cell density to 0.4×10⁶/ml, culture for 24 hours and then perform transfection. The cell density on the day of transfection is approximately 0.8×10⁶/ml.

4.Transfect the plasmid into 5 ml of cell culture medium using a specially prepared transfection reagent.

5.Collect the supernatant, purify it, and detect the expression situation by SDS-PAGE.

6.Analysis of the small sample expression results: The protein is expressed in the supernatant, and secretory expression and purification can be continued.

Large-scale expression and protein purification

1.Cultivate 293f cells in large quantities in a 37°C constant temperature incubator with 5% CO₂at 120 rpm.

2.One day before transfection, collect the cells by centrifuging at 1000 rpm for 5 minutes, resuspend them in 293f expression medium, adjust the cell density to 0.4×10⁶/ml, culture for 24 hours and then perform transfection. The cell density on the day of transfection is approximately 0.8×10⁶/ml, and the total volume is greater than 100 ml.

3.Transfect the plasmid into 100 ml of cell culture medium using a specially prepared transfection reagent.

4.Collect the supernatant, purify it with Protein A, and detect the expression situation by SDS-PAGE.

5.Analysis of the large-scale expression results: The target protein is expressed in the soluble supernatant. The total amount is 15 mg. The expected molecular weight is 35 kDa. The concentration is 1 mg/ml. The purity is greater than 90%, and there is no degradation during the expression process.

Alpaca immunization

Immunization

1.Prepare healthy alpacas of appropriate age for immunization.

2.Conduct immunization and booster immunization according to the immunization schedule. A total of 10 mg of protein is injected. Freund's complete adjuvant is used for the primary injection, and Freund's incomplete adjuvant is used for the booster injection.

3.Take out the antigen that has been aliquoted and stored at -20 °C and dissolve it at 4 °C.

4.Emulsify it on a mixer. (Emulsification requirement: no stratification after standing for 2 days).

5.Load the needle and load the emulsified antigen into a disposable syringe.

6.Inject the alpaca on both the left and right sides near the cervical lymph nodes.

Antiserum verification
Arrange blood collection according to the immunization schedule. After obtaining P1 serum, serum verification is required. Select the corresponding detection antigen according to the project design and conduct ELISA quality verification. (The method refers to the ELISA operation procedure).

1.Coat the antigen at 50 ng per well using 0.1 M NaHCO₃ coating buffer. Dilute the serum into 8 gradients in total using MPBS with serial dilution ratios of 1:1000, 1:2000, and 1:4000.

2.Incubate with the anti-alpaca serum secondary antibody diluted at 1:10000, develop color, and read the values.

3.If the color development is qualified when the dilution ratio exceeds 1:32000, then extract PBMC.

Construction of phage library

RNA extraction

1. Take out the PBMC samples from the -80 °C freezer, aliquot them into 1.5 mL tubes at 1.5 mL per tube, add 200 μL of chloroform to each tube, vigorously shake for 15 seconds on a shaker, and then let it stand at room temperature for 5 minutes.
2. Pre-cool the centrifuge to 4 °C, centrifuge at 12,000 g for 15 minutes. After centrifugation, stratification will occur. Carefully transfer the transparent layer on the topmost layer to new 1.5 mL centrifuge tubes that are free of RNase and DNase using a pipette. Add 250 μL of isopropanol to each tube, mix thoroughly by inverting several times, and then let it stand at room temperature for 10 minutes.
3. Use the RNA extraction kit RC112 product of Vazyme Biotech Co., Ltd. for the subsequent steps. The steps are as follows:
4. Transfer all the mixtures in step 2 to FastPure RNA Columns III (FastPure RNA Columns III has been placed in the collection tube), centrifuge at 12,000 rpm for 30 seconds, and discard the filtrate.
5. Add 700 μL of Buffer RW1 to FastPure RNA Columns III, centrifuge at 12,000 rpm for 30 seconds, and discard the filtrate.
6. Add 700 μL of Buffer RW2 (with anhydrous ethanol already added) to FastPure RNA Columns III, centrifuge at 12,000 rpm for 30 seconds, and discard the filtrate.
7. Add 500 μL of Buffer RW2 (with anhydrous ethanol already added) to FastPure RNA Columns III, centrifuge at 12,000 rpm for 2 minutes. Carefully take out the adsorption column from the collection tube to avoid contact with the filtrate and causing contamination.
8. Put FastPure RNA Columns III back into the collection tube, centrifuge empty at 12,000 rpm for 1 minute to prevent ethanol contamination.
9. Carefully transfer the adsorption column to new 1.5 mL RNase-free Collection Tubes. Slowly drip 50 μL of RNase-free ddH2O into the center of the adsorption column, let it stand at room temperature for 1 minute, and then centrifuge at 12,000 rpm for 1 minute to elute the RNA.
10. Take 1 μL of the above total RNA for 1% agarose gel electrophoresis to detect the integrity of the total RNA. Meanwhile, take 2 μL to measure the concentration of RNA and the A260/A280 ratio using a nucleic acid concentration meter and record the results.
11. Refer to the kit for the reverse transcription steps. Use the reverse transcription kit of Vazyme Biotech Co., Ltd. Store the cDNA after reverse transcription at -20 °C.

RNA reverse transcription

Use the Oligo dT Primer in the Vazyme RNA reverse transcription kit (HiScript II Q Select RT SuperMix for qPCR R232) as the primer for the total RNA samples obtained in 3.2.1. Prepare the reaction solution in a 1.5 mL centrifuge tube according to the proportion in the following table. When the amount of total RNA is greater than 5 μg, expand the reaction system in the same proportion.

| Reagent | Usage amount |
| --- | --- |
| Oligo dT Primer（50μM） | 1μL |
| Total RNA | 5μg |
| ddH_2_O | Up to 8 μL |

Put the centrifuge tube containing the above reaction solution into a metal heater, react at 65 °C for 5 minutes to denature the RNA. After the reaction is completed, place it on ice to cool rapidly. Then prepare the reaction solution in the above 1.5 mL centrifuge tube according to the proportion in the following table. When the total volume in the tube is greater than 10 μL, expand the reaction system in the same proportion.

| Reagent | Usage amount |
| --- | --- |
| Reaction solution | 8μL |
| 2×RT Mix | 10μL |
| HiScript II Enzyme Mix | 2μL |

Mix slowly, then put the centrifuge tube containing the above reaction solution into a metal heater, react at 50 °C for 60 minutes, then at 85 °C for 2 minutes, and cool on ice. This is the cDNA after reverse transcription of the total RNA. Store it at -20 °C.

Design primers and amplify VHH genes

Use cDNA as the template and use the 2×Phanta Max Master Mix of Vazyme Biotech Co., Ltd. to amplify the first round of VHH by PCR. Configure the PCR reaction system according to the following table.

| Reagent | Usage amount |
| --- | --- |
| 2×Phanta Max Master Mix | 10μL |
| Primer1 | 0.4μL |
| Primer2 | 0.4μL |
| cDNA | 0.6μL |
| ddH2O | Up to 20μL |

Conduct the PCR reaction: 95 °C for 3 minutes; 95 °C for 15 seconds, 56 °C for 15 seconds, 72 °C for 20 seconds, 32 cycles; 72 °C for 5 minutes; 4°C for infinity.

After the reaction is completed, take 20 μL of the PCR product for 1% agarose gel electrophoresis and cut out the band with a fragment size of 600 bp.

Gel extraction

Use the DNA purification and recovery kit of OMEGA to purify the DNA in the PCR reaction solution. Conduct the purification according to the kit instructions. The steps are as follows: 1. Transfer the gel block containing the target fragment to a 1.5 mL centrifuge tube (the centrifuge tube has already been weighed). Weigh the gel block to obtain its weight, approximately determine its volume, add an equal volume of XP2 Binding Buffer, incubate in a water bath at 50 - 60 °C for 7 minutes or until the gel is completely melted, and shake or vortex the mixture every 2 - 3 minutes. 2. Take a HiBind® DNA Mini binding column and install it in a 2 mL collection tube. Transfer all the DNA/gel melting solution obtained in step 3 to the HiBind® DNA Mini binding column. 3. Centrifuge at 10,000 x g for 1 minute at room temperature, discard the filtrate in the collection tube, and put the column back into the 2 mL collection tube. 4. If the volume of the DNA/gel melting solution exceeds 700 μL, only transfer 700 μL to the HiBind® DNA Mini binding column at a time, and repeat steps 3 until all the DNA/gel melting solution is bound to the column. 5. Discard the filtrate in the collection tube, and put the HiBind® DNA Mini binding column back into the 2 mL collection tube. Transfer 300 μL of XP2 Binding Buffer to the column, centrifuge at the maximum speed (≥13,000) for 1 minute at room temperature, and discard the filtrate. 6. Put the HiBind® DNA Mini binding column back into the 2 mL collection tube. Transfer 700 μL of SPW Buffer (already diluted with anhydrous ethanol) to the HiBind® DNA Mini binding column. Centrifuge at 10,000 x g for 1 minute at room temperature, and discard the filtrate. 7. Repeat step 6. 8. Put the HiBind® DNA Mini binding column back into the 2 mL collection tube. Centrifuge at ≥13,000 x g for 2 minutes at room temperature to dry the residual liquid in the matrix of the HiBind® DNA Mini binding column. 9. Install the HiBind® DNA Mini binding column on a clean 1.5 mL centrifuge tube, add 15 - 30 μL of Elute Buffer to the matrix of the HiBind® DNA Mini binding column, let it stand at room temperature for 1 minute, and centrifuge at 13,000 x g for 1 minute to elute the DNA. 10. Take 2 μL to measure the concentration of DNA and the A260/A280 ratio using a nucleic acid concentration meter and record the results. 11. Use the first-round product as a template for the second-round PCR reaction. The PCR system and procedure are the same as those of the first round. The gel band is about 350 bp. The purification steps are the same as mentioned to obtain the final VHH product.

Ligation of VHH fragments and vectors

Use Goldgate to ligate VHH and the phagemid vector. Prepare the ligation reaction system according to the following table:

| Reagent | Usage amount |
| --- | --- |
| T4 DNA Ligase | 10 μL |
| BsaIHF-V2 | 10 μL |
| 10×T4 DNA Ligase buffer | 50 μL |
| pComb3XSS-2 vector | 10μg |
| VHH | 2100ng |
| ddH2O | Up to 500μL |

The reaction conditions are as follows: incubate at 37 °C for 10 minutes; then conduct 40 cycles of incubation at 37 °C for 5 minutes and at 16 °C for 5 minutes; incubate at 55 °C for 10 minutes; store at 4 °C.

Recover the product by sodium acetate-ethanol precipitation, wash it twice with 75% ethanol, and dissolve it in 10% glycerol.

Electroporation of ligation products

1. Take out the commercial TG1 competent cells from the -80 °C freezer and thaw them in an ice bath for 10 minutes. 2. Add the purified ligation products on ice and let them stand for 15 minutes. 3. Transfer the mixture into a 2-mm electroporation cuvette and wipe off the water droplets on the outer wall of the electroporation cuvette. 4. Set the voltage at 2.5 kV and conduct an electric shock for 5 ms. 5. Immediately add 2 mL of pre-warmed medium for recovery and incubate at 37 °C with 220 rpm for 1 hour. 6. Dilute the bacterial solution to 10⁻⁵, 10⁻⁶, and 10⁻⁷, and spread it on 2YT-AG plates for overnight culture to detect the library size and diversity. 7. Spread the remaining bacterial solution on large plates and culture it overnight at 37 °C.

Analysis of library diversity

| Reagent | Usage amount |
| --- | --- |
| 2×Taq Mix | 5μL |
| Primer1 | 0.2μL |
| Primer2 | 0.2μL |
| cDNA | 0.2μL |
| ddH_2_O | Up to 10μL |

1. Randomly pick several single-clone bacteria on the plate and inoculate them into 1 mL of 2YT liquid medium (containing Amp resistance) in a centrifuge tube. Cultivate at 37 °C for 3 hours. Prepare the PCR reaction according to the following table and conduct

single-colony PCR.

Conduct the PCR reaction: 95 °C for 3 minutes; 95 °C for 15 seconds, 56 °C for 15 seconds, 72 °C for 20 seconds, 32 cycles; 72 °C for 5 minutes; 4 °C for infinity.

1. Perform 1% agarose gel electrophoresis on the single-colony PCR products. Colonies with the target band having a fragment size of 500 bp are identified as positive clones. 3. Randomly select several clones from the positive clones detected by colony PCR and send them for sequencing. Translate the light chain antibody fragments in the sequencing results into amino acid sequences and then conduct sequence alignment to detect the sequence diversity in the bacterial library.

Preparation of phage antibody library and antigen panning

Preparation of phage antibody library 1. Add 30 OD glycerol bacteria to 300 mL of 2YT (A&G), and incubate at 37 °C with 220 rpm/min until the OD value reaches 0.4 - 0.6. 2. Add M13K07 (3×10¹²), shake well, let it stand at 37 °C for 30 minutes, and then incubate at 37 °C with 220 rpm/min for 1 hour. 3. Centrifuge at 5000 rpm for 5 minutes and discard the old medium. 4. Resuspend the pellet after centrifugation with an equal volume of 2YT (A&K), and incubate overnight at 30 °C with 220 rpm/min. 5. Aliquot into tubes at 35 mL per tube, and centrifuge at 6000 rpm for 10 minutes. 6. Keep the supernatant, transfer it to clean tubes, and add 1/5 volume of PEG-NaCl. Let it stand at 4 °C for 2 hours. 7. Centrifuge at 8000 g for 10 minutes at 4 °C. 8. Discard the supernatant, keep the pellet, dissolve the pellet with 10 mL of PBS, and centrifuge at 8000 g for 5 minutes at 4 °C. 9. Keep the supernatant, transfer it to clean tubes, and add 1/5 volume of PEG-NaCl. Let it stand at 4 °C for 60 minutes. 10. Centrifuge at 8000 g for 10 minutes at 4 °C. 11. Discard the supernatant, keep the pellet, and dissolve the pellet with an appropriate amount of PBS. 12. Measure the titer.

Antigen panning and amplification 1. Coat the antigen in the immunization tube at 30 μg per tube, add 0.1 M NaHCO₃ to make up to 2 mL, and let it stand at 37 °C for 2 hours. 2. After pouring out the antigen, wash with PBST for 3 times, add 5 mL of 4% milk in PBS for blocking, and let it stand at 37 °C for 1 hour. 3. After pouring out the 4% milk in PBS, wash with PBST for 3 times, add 1×10¹² phages for panning, and let it stand at 37 °C for 1 hour. 4. After pouring out the phages, wash with PBST for 10 times. 5. Add 1 mL of Gly-HCl with pH 2.0 and shake at room temperature for 8 minutes. 6. Add 200 μL of Tris-HCl with pH 9.5 for neutralization to obtain the elution solution. 7. Except for measuring the titer of the elution solution, use the rest to infect 5 mL of TG1 and let it stand at 37 °C for 0.5 hour. 8. Add 2YTAG to make up to 20 mL, incubate the bacteria at 37 °C with 220 rpm for 4 to 6 hours until the OD₆₀₀ is about 0.5. 9. Add the helper phage M13K07 according to MOI = 20, and let it stand at 37 °C for 0.5 hour. 10. Centrifuge to collect the pellet and transfer it to 150 mL of 2YTAKI medium, and incubate overnight at 28 °C with 220 rpm. 11. Collect the phages by the method mentioned.

ELISA verification

1. After the phages obtained from the third round of panning infect TG1, pick single clones and inoculate them into 4 - 5 96-well plates for culturing bacteria. 2. After the OD₆₀₀ of the bacterial solution reaches about 0.5, save the bacterial solution, then add the helper phage M13K07, let it stand at 37 °C for 0.5 hour, and induce phages overnight at 28 °C with 220 rpm. 3. Coat the antigen onto the ELISA plate at 50 ng per well using NaHCO₃, and let it stand at 4 °C overnight. 4. Wash with PBST for 3 times, then add 4% MPBS for blocking, and block at 37 °C for 1 hour. 5. Wash with PBST for 3 times, then add 50 μL each of the phages and 4% MPBS, and incubate at 37 °C for 1 hour. 6. Wash with PBST for 4 times, then add 100 μL of the diluted anti-M13-HRP antibody, and react at 37 °C for 1 hour. 7. Wash with PBST for 5 times, then add TMB chromogenic solution for color development, terminate with sulfuric acid, and read the absorbance at 450 nm on a microplate reader. 8. Keep the phage supernatants with positive color development for flow cytometry detection.

Flow cytometry verification

1. Prepare the cells required for flow cytometry and phage supernatants. Each phage sample requires 1×10⁵ positive cells. 2. At 4 °C, use 3% BSA-PBS to block the cells and phages for 30 minutes. 3. Incubate the phages and positive cells at 4 °C for 30 minutes, and wash with PBS for 3 times. 4. Add the labeled antibody and incubate at 4 °C for 30 minutes, and wash with PBS for 3 times. 5. Add the corresponding fluorescent secondary antibody and incubate at 4 °C for 30 minutes, and wash with PBS for 3 times. 6. Perform detection on the flow cytometer. 7. Sequence the flow cytometry positive samples.

**Experimental results**

Results of eukaryotic protein expression verification

The protein was expressed both in the supernatant and inside the cells.


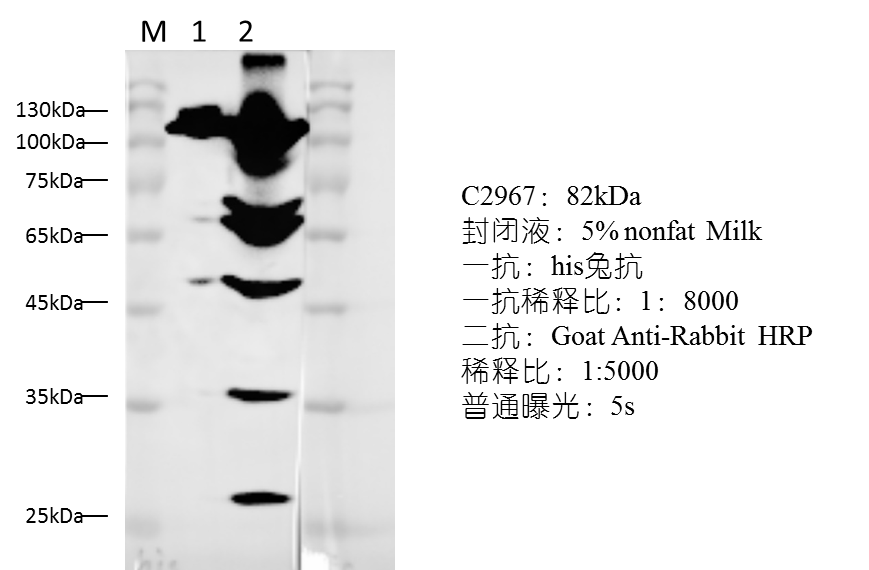


1: Eukaryotic protein C2967 (indicated this experiment) (enriched in the nickel column supernatant, 10 μL/lane), 2: Eukaryotic protein C2967 (cell lysate, 10 μL/lane)

Immunization results

The eukaryotically expressed Human PSMA protein was used as an antigen and injected into one alpaca. Freund's complete adjuvant was used for the first primary injection, and Freund's incomplete adjuvant was used for subsequent booster injections. They were thoroughly mixed with an equal volume of the antigen before injection.

Antigen information for the C2967 project

| Number | Protein | Size | Positive cells | Negative cells |
| --- | --- | --- | --- | --- |
| C2967 | PSMA | 707aa,90~120kDa | Lncap | PC-3 |

After the last few immunizations, the PBMC and serum of the alpaca were collected. The corresponding antigen at 50 ng was coated in ELISA strips and then blocked. The serum was diluted in MPBS at ratios of 1:100 and 1:1000. 100 μL of the diluted serum was added to the first well, and serial dilutions were made for subsequent wells. Incubate with the HRP-labeled alpaca secondary antibody, and then develop color using TMB.


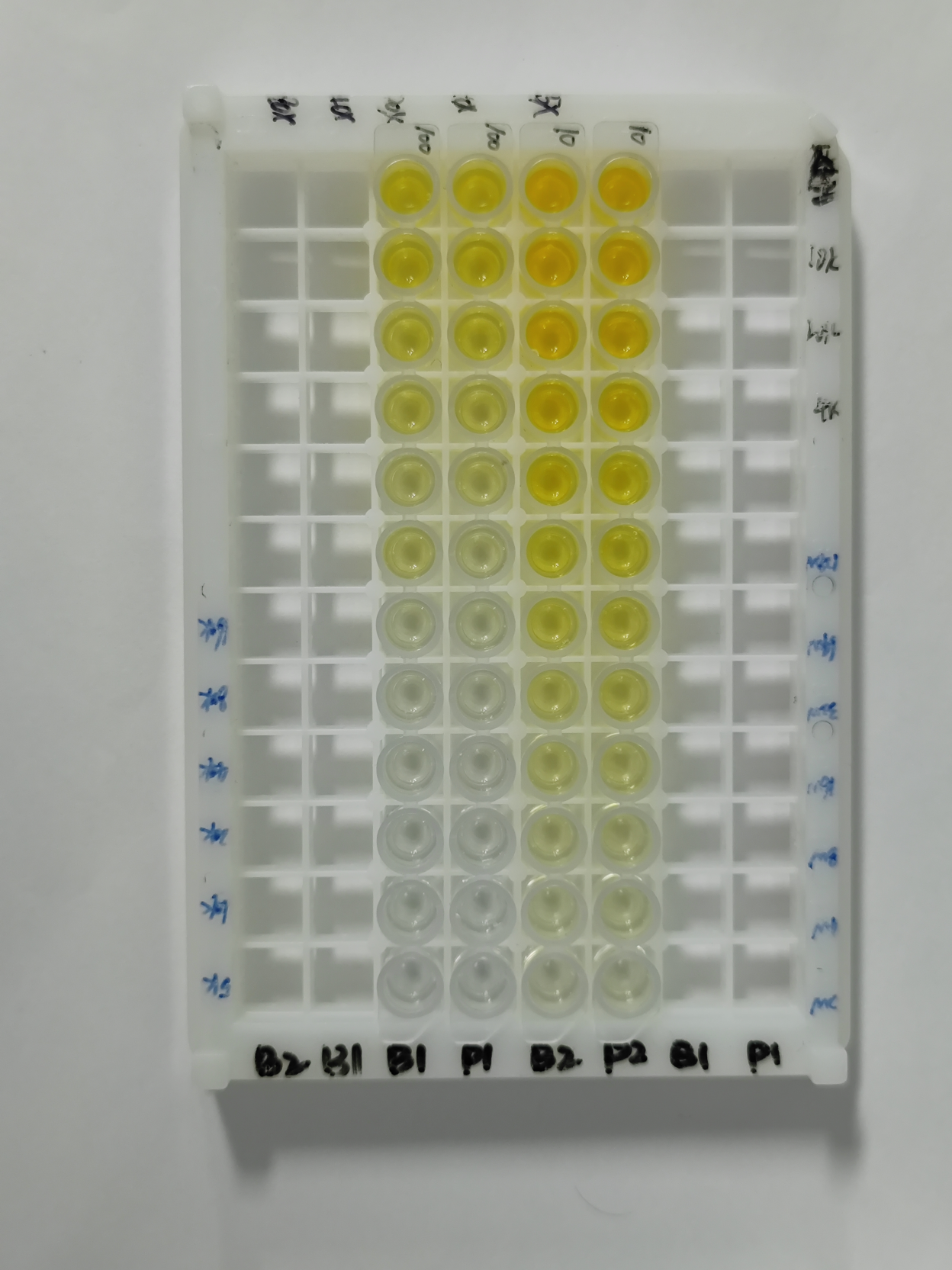


Results of protein serum titer detection

Construction of phage library

Amplification of VHH fragments

The PCR products were separated by TBE electrophoresis, and then the products were recovered by gel extraction. A 750 bp band was cut out in the first round of PCR, and a band of about 350 bp was cut out in the second round of PCR.


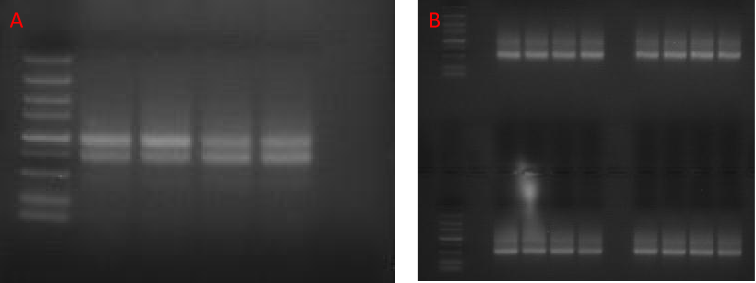


Gel electrophoresis results of the first and second rounds of PCR Figure A: Gel electrophoresis results of the first-round PCR products. The marker is Vazyme DL5000 DNA Marker, and the bands are 5 k, 3 k, 2 k, 1.5 k, 1 k, 750 bp, 500 bp, 250 bp, and 100 bp respectively. Figure B: Gel electrophoresis results of the second-round PCR products. The marker is the same as that in Figure A.

Identification of library size

RNA was extracted from the third batch of returned PBMCs, reverse transcribed and used as a template to amplify VHH fragments. These fragments were then ligated to the phagemid vector and electroporated into TG1. The ligation products were electroporated into TG1 multiple times, and finally a library size of 1.2×10⁸ cfu was obtained. Primers on the vector were used to detect the positive rate of the library and conduct sequencing. It was found that the insertion rate of the library was 91%.


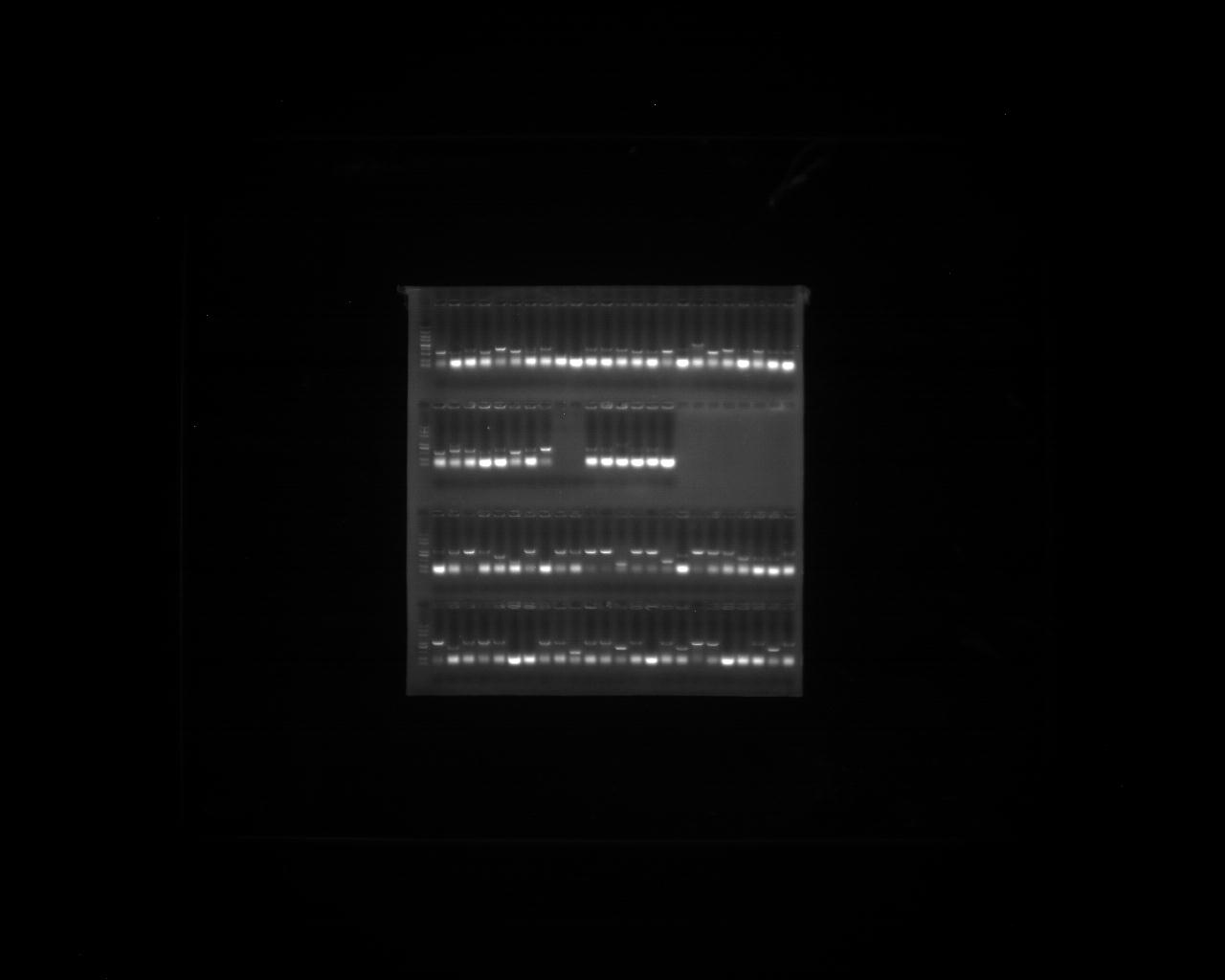


Detection of library insertion rate

Panning results

Library amplification and panning

The library bacteria were rescued by the helper phage to obtain an antibody library, resulting in a phage antibody library of approximately 9.0×10¹¹. Five antigens were used for panning, and the phages eluted in the third round were verified by ELISA. The positive phages were then detected by flow cytometry. The statistics of the phage titers for each panning and amplification are shown in the following table. Results of the first panning:

| Project number | Input | Dilution factor 10ⁿ | | | Output | Protein coating amount |
| --- | --- | --- | --- | --- | --- | --- |
|  |  | 4 | 5 | 6 | pfu/mL |  |
| C2967-Panning1 | 1.1x10^12^ | / | 102~~/28~~ | 11/12 | 1.1x10^7^ | 30ug |

Results of the first amplification

| Project number | Dilution factor 10ⁿ | | | pfu/mL |
| --- | --- | --- | --- | --- |
|  | 9 | 10 | 11 |  |
| C2967-1st amplification | / | 114/83 | 20/18 | 2.0x10^12^ |

Results of the second panning

| Project number | Input | Dilution factor 10ⁿ | | | Output | Protein coating amount |
| --- | --- | --- | --- | --- | --- | --- |
|  |  | 4 | 5 | 6 | pfu/mL |  |
| C2967-Panning2 | 1.0x10^12^ | 991/886 | 101/92 | 14/9 | 9.0x10^6^ | 30ug |

Results of the third panning

| Project number | Input | Dilution factor 10ⁿ | | | Output | Protein coating amount |
| --- | --- | --- | --- | --- | --- | --- |
|  |  | 4 | 5 | 6 | pfu/mL |  |
| C2967-Panning3 | 1.0x10^12^ | 701/656 | 91/88 | 10/7 | 7.0x10^6^ | 30ug |

ELISA results


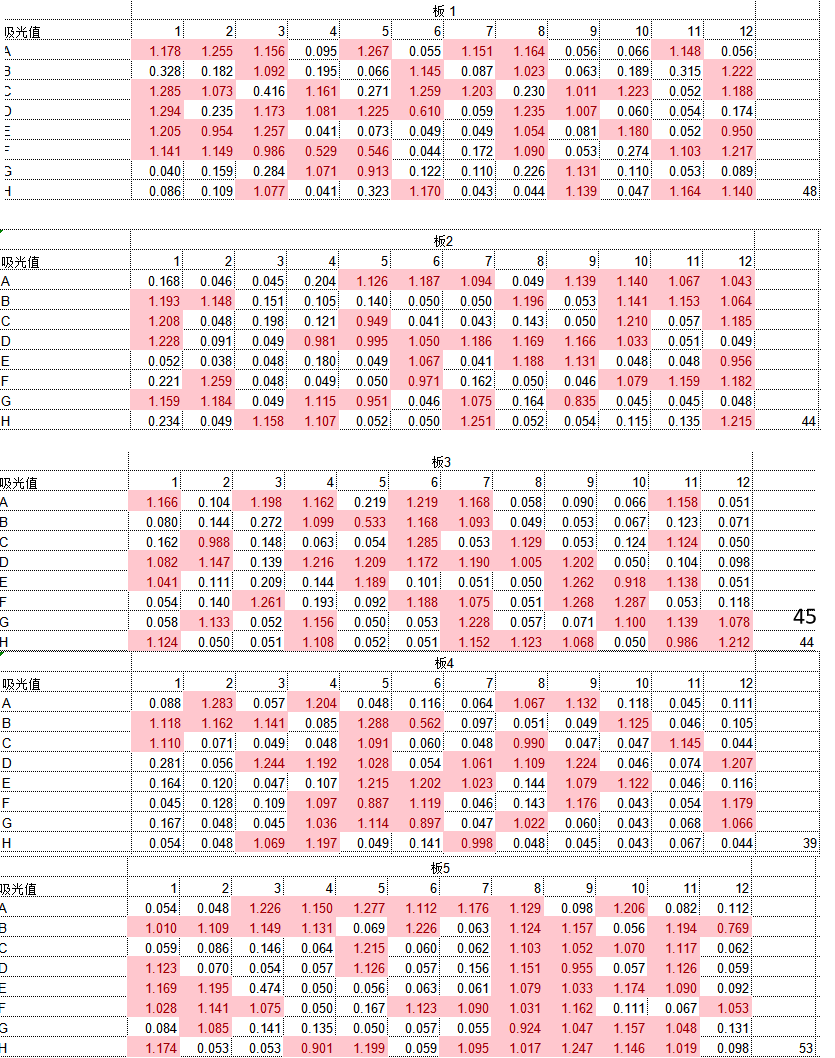


Select the items with high ELISA readings (the items marked in red) for flow cytometry verification. Nine colonies with positive results were selected and sequenced, and nine VHH clones with known sequences were cloned into a plasmid vector to produce anti-PSMA VHHs

**Measurement of the affinity of the PSMA-directed VHH sequence 1H5**

**Basic information**

Instruments：Octet R8，Startorius BioAnalytical Instru ments inc;

Consumables: 1.5 ml EP tubes, 15 ml centrifuge tubes, 96-well black opaque plates, and a certain number of pipette tips of various specifications;

Chip: NTA (Manufacturer: Sartorius, Article No.: 18-5101);

Buffers: 1× PBS, PBST;

Reagents: Recombinant Human PSMA Protein, 20 μg, ~100 KDa; 1H5-Biotin, 3.4 mg/ml, ~15 KDa.

**Interaction experiment**

Since the Recombinant Human PSMA Protein carries a His tag, it can be specifically captured by the NTA chip. After the signal reaches 1.5 nm, it will bind with 1H5-Biotin. The schematic diagram of the principle is as follows:


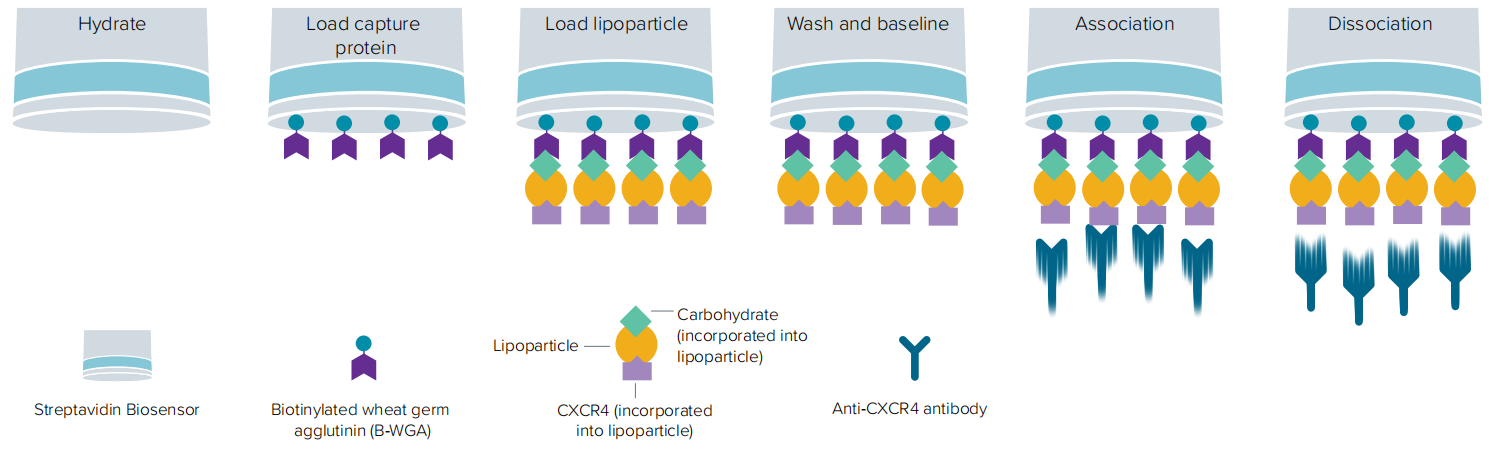


Reconstitute the Recombinant Human PSMA Protein with PBS to a concentration of 0.5 mg/ml, and then dilute it with 1× PBS to a concentration of 5 μg/ml. Fix it for 600 seconds.

Dilute the 1H5-Biotin with PBST buffer to a concentration of 1 μM.

Add the samples to the 96-well plate in the following order.


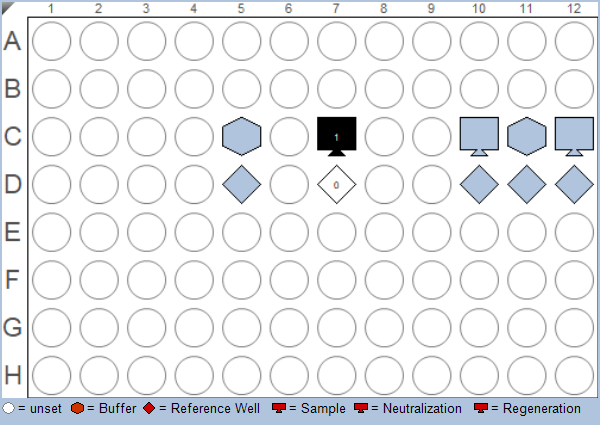


Among them:
Add PBST to columns 5 and 11;
Add 1H5-Biotin to column 7, and add PBST to well D7 for zero concentration subtraction;
Add nickel chloride to column 10 for chip regeneration.
Add glycine·HCl to column 12 for chip regeneration.
Program setting

| Assay Step Number | Step Data Name | Step Type | Assay Time |
| --- | --- | --- | --- |
| 1 | Baseline | Baseline | 60 |
| 2 | Association | Association | 200 |
| 3 | Dissociation | Dissociation | 300 |
| 4 | Custom | Custom | 3 |
| 5 | Custom | Custom | 3 |
| 6 | Custom | Custom | 3 |
| 7 | Custom | Custom | 3 |
| 8 | Custom | Custom | 3 |
| 9 | Custom | Custom | 3 |
| 10 | Baseline2 | Baseline | 120 |

Chip position


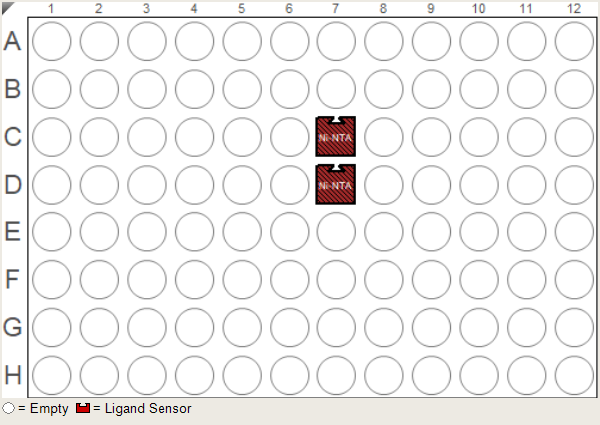


A total of 2 chips were used in this experiment and placed in column 7. The chips were wetted 10 minutes before the start of the experiment. The wetting buffer was 1× PBS.

**Results**

After the program finished running, the following raw data were obtained (see the figure below).


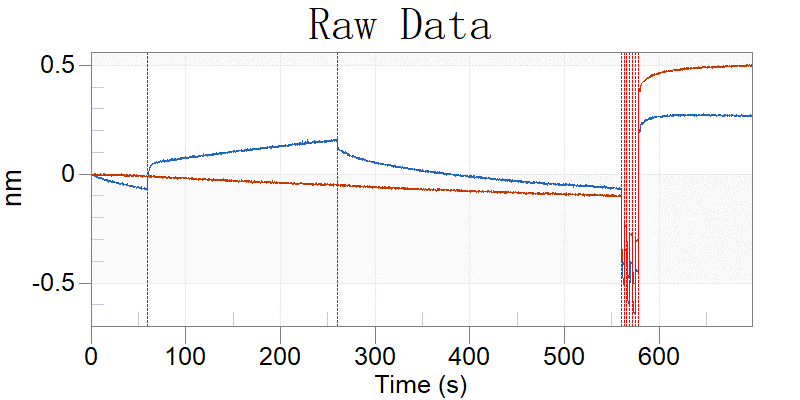


Processed data：


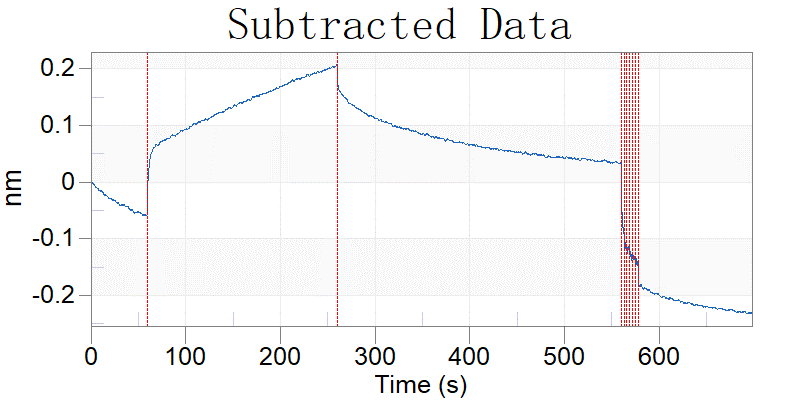


The meanings represented by the various colored lines in the figure are as follows:

|  | Signals and data of the interaction between the immobilized chip and 1H5-Biotin |
| --- | --- |
|  | Signals and data of the interaction between the immobilized chip and PBST |

Basic analysis and judgment of the data:
All the immobilization signals were at 1.5 nm, and the binding signal between the Recombinant Human PSMA Protein and 1H5-Biotin was relatively strong.

Software processing:
Software version: fortebio data analysis 12.0
Perform the "align" processing on the data.
X ALIGN 55 - 59.8S
Y ALIGN DISSOCIATION, and the following data were obtained.


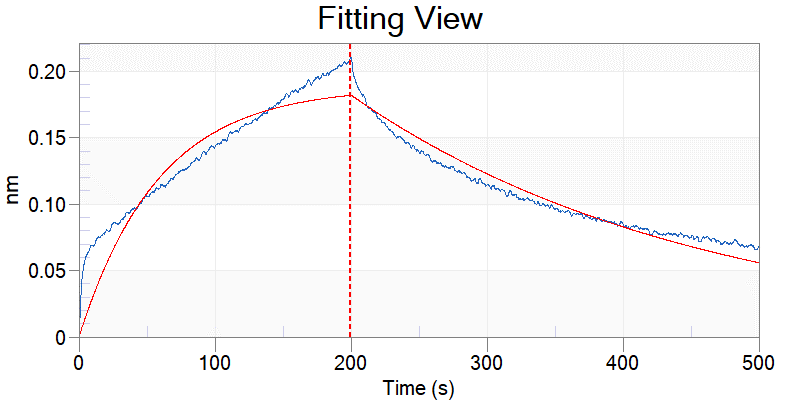


| KD (M) | KD Error | kon(1/Ms) | kon Error | kdis(1/s) | kdis Error | RMax | RMax Error | kobs(1/s) | Req | Req/Rmax(%) | Full X^2 | Full R^2 |
| --- | --- | --- | --- | --- | --- | --- | --- | --- | --- | --- | --- | --- |
| 2.98E-07 | 1.30E-08 | 1.32E+04 | 5.36E+02 | 3.94E-03 | 6.38E-05 | 0.2441 | 0.0049 | 1.72E-02 | 0.1881 | 77.1 | 0.0674 | 0.92 |

**Conclusion**

Recombinant Human PSMA Protein binds to 1H5-Biotin, with a KD value of 2.98×10^-7^.

**Construction of anti-PSMA Ab with anti-PSMA VHH and Fc and production of anti-PSMA Ab**

**General Procedure**

1. Protein Expression:

1) Target cDNA was inserted into baculovirus vector followed by the generation of the recombinant baculovirus according to the manufacturer’s manual.

2) Recombinant baculovirus was amplified in cells to prepare high titer virus stocks.

3) For protein expression, cells were infected with recombinant baculovirus following standard protocols and target protein was expressed under optimal conditions.

2. Protein Purification and Analysis

1) Cell pellets were collected via centrifugation and resuspended in homogenizing buffer.

2) Pellets were homogenized and centrifuged to remove cellular debris.

3) The lysate supernatant was collected and loaded onto an affinity purification column.

4) Target protein was eluted from the column using elution buffer. Fractions containing the protein-of-interest were pooled and underwent buffer exchange into formulation buffer.

5) Protein concentration of the final product was determined by UV or BCA assays.

6) The purity of the final product was analyzed by SDS-PAGE.

1. **The amino acid sequence of the anti-PSMA Ab protein with a theoretical molecular weight of 39.57 kDa**

**SP**QVQLVESGGGLVQPGGSLRLSCAASGFTLDYYTIGWFRQASGKEREWVSSLSSSDGSSYYADSVKGRFTISRDNAKNTVHLQMNSLKPEDTAVYYCAAAESDTMVVAATVNGMDYWGKGTLVTVSSEPKSCDKTHTCPPCPAPELLGGPSVFLFPPKPKDTLMISRTPEVTCVVVDVSHEDPEVKFNWYVDGVEVHNAKTKPREEQYNSTYRVVSVLTVLHQDWLNGKEYKCKVSNKALPAPIEKTISKAKGQPREPQVYTLPPSRDELTKNQVSLTCLVKGFYPSDIAVEWESNGQPENNYKTTPPVLDSDGSFFLYSKLTVDKSRWQQGNVFSCSVMHEALHNHYTQKSLSLSPGK

**SP**: signal peptide；The labeled sequence in blue was Fc region of human IgG1

1. **Construction of eukaryotic expression vectors**

The sequence of plasmid SP-VHH-IgG1 Fc-VB5 and chromatogram were as follows:

SPCAGGTGCAGCTCGTGGAGTCTGGGGGAGGCTTGGTGCAGCCTGGGGGGTCTCTGAGACTCTCCTGTGCAGCCTCTGGATTCACTTTGGATTATTATACCATAGGCTGGTTCCGCCAGGCCTCAGGGAAGGAGCGCGAGTGGGTCTCATCTCTTAGTAGTAGTGATGGTAGCTCATACTATGCAGACTCCGTGAAGGGCCGATTCACCATCTCCAGAGACAACGCCAAGAACACGGTACATCTGCAAATGAACAGTCTGAAACCCGAGGACACGGCCGTTTATTACTGTGCAGCAGCAGAGTCGGATACTATGGTGGTAGCGGCTACGGTCAACGGCATGGACTACTGGGGCAAAGGGACCCTGGTCACCGTCTCCTCAGAGCCCAAATCTTGTGACAAAACTCACACATGCCCACCGTGCCCAGCACCTGAACTCCTGGGGGGACCGTCAGTCTTCCTCTTCCCCCCAAAACCCAAGGACACCCTCATGATCTCCCGGACCCCTGAGGTCACGTGCGTGGTGGTGGACGTGAGCCACGAAGACCCCGAGGTCAAGTTCAACTGGTACGTGGACGGCGTGGAGGTGCATAATGCCAAGACAAAGCCGCGGGAGGAGCAGTACAACAGCACGTACCGTGTGGTCAGCGTCCTCACCGTCCTGCACCAGGACTGGCTGAATGGCAAGGAGTACAAGTGCAAGGTCTCCAACAAAGCCCTCCCAGCCCCCATCGAGAAAACCATCTCCAAAGCCAAAGGGCAGCCCCGAGAACCACAGGTGTACACCCTGCCCCCATCCCGGGATGAGCTGACCAAGAACCAGGTCAGCCTGACCTGCCTGGTCAAAGGCTTCTATCCCAGCGACATCGCCGTGGAGTGGGAGAGCAATGGGCAGCCGGAGAACAACTACAAGACCACGCCTCCCGTGCTGGACTCCGACGGCTCCTTCTTCCTCTACAGCAAGCTCACCGTGGACAAGAGCAGGTGGCAGCAGGGGAACGTCTTCTCATGCTCCGTGATGCATGAGGCTCTGCACAACCACTACACGCAGAAGAGCCTCTCCCTGTCTCCGGGTAAATGA,*Signal Peptide*

*
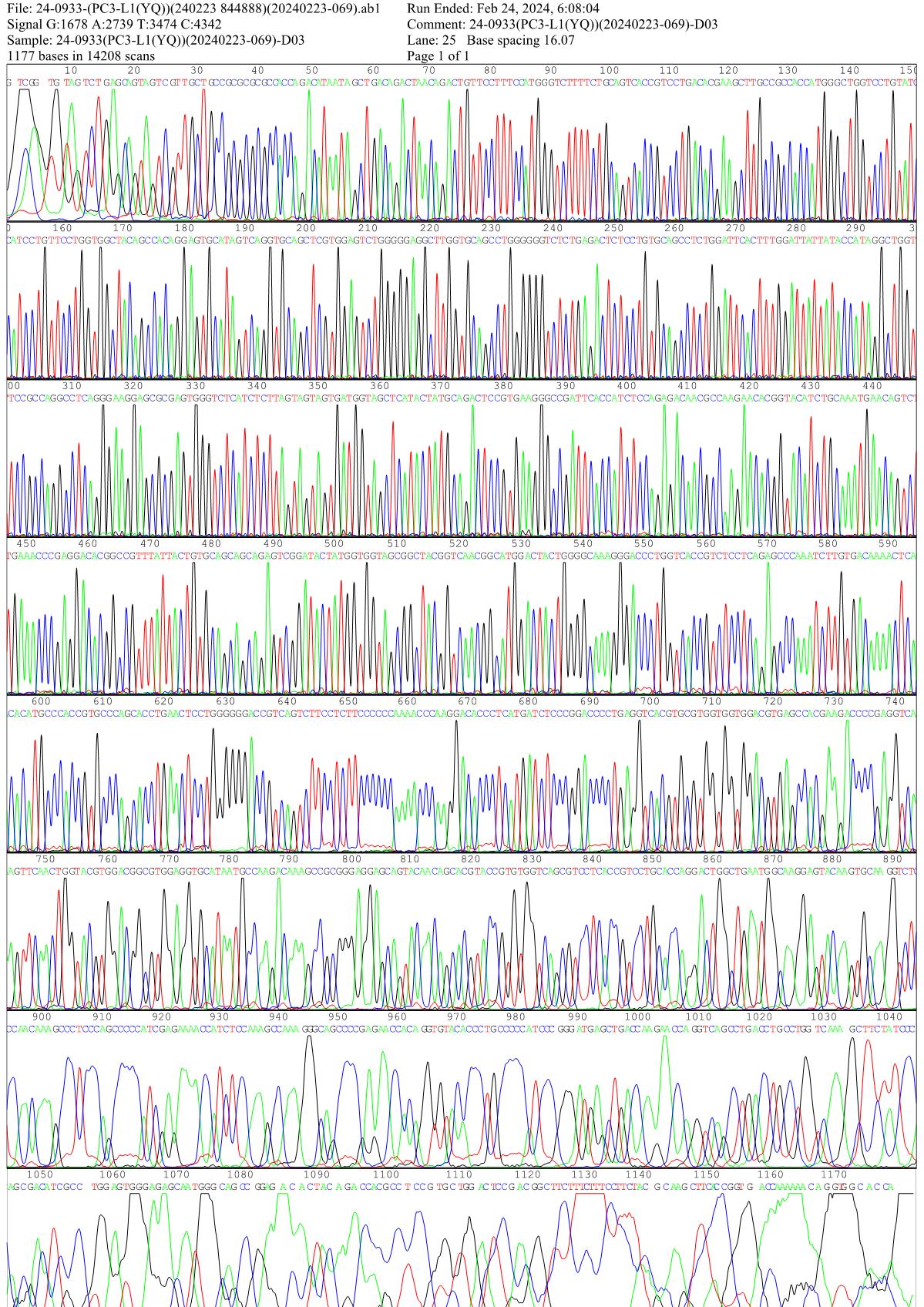
*


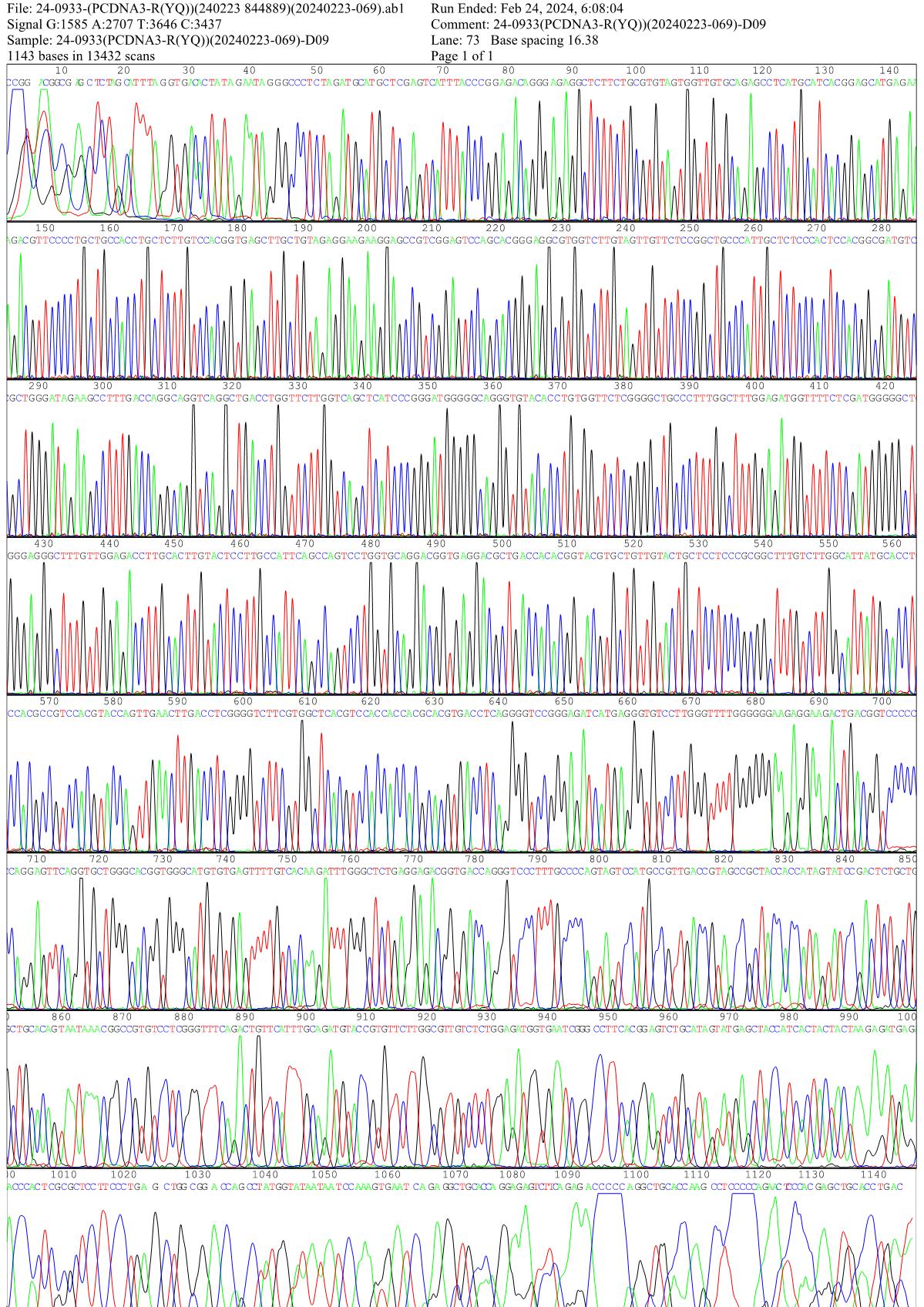


1. **The protein expression and purification**

The HEK293 cells were transduced with VB5 plasmid and the supernatants were collected after seven days culture. The protein A affinity chromatography were used to acquire target protein with purity >90%.


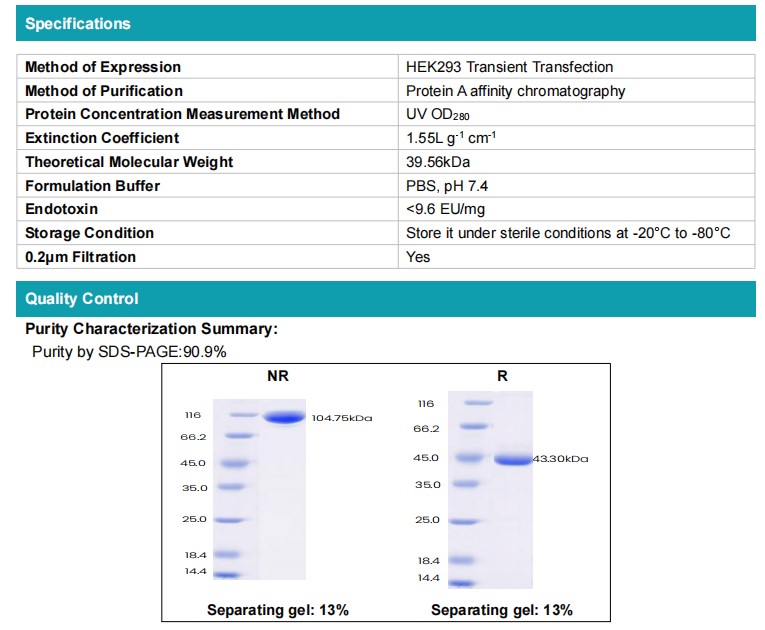


The protein A affinity chromatography showing target protein was purified. The left

was reducing sodium dodecyl sulfate polyacrylamide gel electrophoresis (SDS-PAGE), the left was non-reducing (NR) SDS-PAGE.
