# Supplementary material for: Combined treatment with anti-PSMA antibody and human peripheral blood-derived NK cells for castration-resistant prostate cancer
Source: Front Immunol. 2025 May 21;16:1572676. doi: 10.3389/fimmu.2025.1572676 (PMC12133763; doi:10.3389/fimmu.2025.1572676)

**Supplementary material 3**

**Patient information**

Gender：Male

Ethnicity：East Asian

Age at sampling：In his seventies

TNM Status：T3N1M1

Disease Metastasis Status：Metastatic

Disease Metastasis Sites：bones, lung

Treatment History：Long-term androgen deprivation therapy (ADT) combined with radiotherapy

Tissue：Prostate

Cancer Type：Prostate Carcinoma

Tissue Status：Metastasis

Sample Site：Pleural effusion


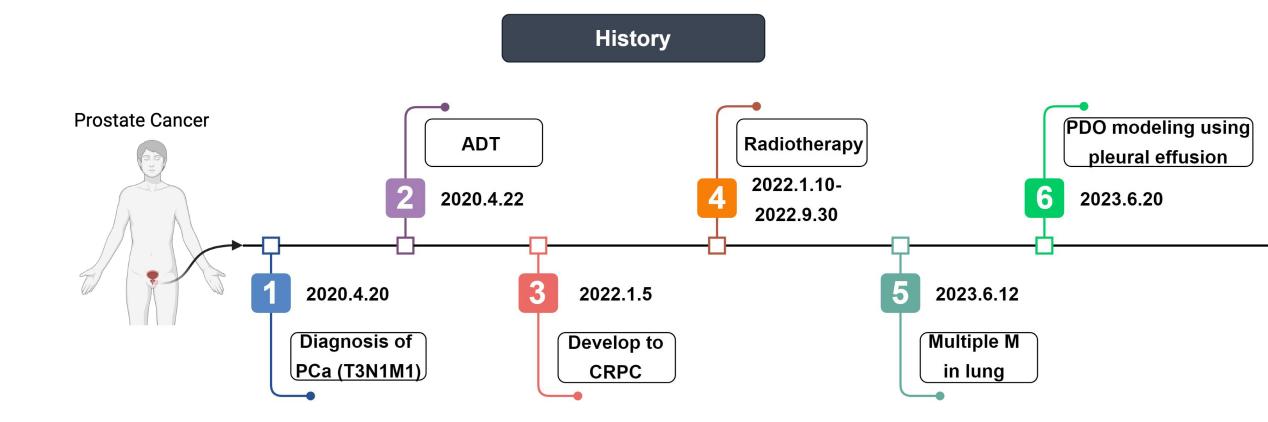

Supplement: Supplementary file 3 [file DataSheet3.docx]
