# Supplementary material for: Combined treatment with anti-PSMA antibody and human peripheral blood-derived NK cells for castration-resistant prostate cancer
Source: Front Immunol. 2025 May 21;16:1572676. doi: 10.3389/fimmu.2025.1572676 (PMC12133763; doi:10.3389/fimmu.2025.1572676)
Supplement: Supplementary file 4 [file DataSheet4.docx]

**Table1 Summary of the properties of the human prostate cancer cell and normal prostate epithelial cell lines**

| Cell Line | Site of Origin | PSA secretion | AR expression | PSMA Expression | Androgen Dependency |
| --- | --- | --- | --- | --- | --- |
| LNCaP | Lymph node metastasis of prostate cancer patient | High - express and can be up - regulated by androgen | High - express | Express | Dependent |
| PC - 3 | Bone metastasis of prostate cancer | None or very low expression | None or extremely low expression | Not expressed | Independent |
| C4 - 2 | Metastatic left supraclavicular lymph node of a prostate cancer patient (sub - line of LNCaP) | Express PSA, regulated by androgen receptor - related pathways | Express, with Src kinase over - expression affecting its trans - activation function | Generally considered to be expressed | Androgen - independent |
| 22RV1 | Derived from a xenograft after castration - induced regression and relapse of the parental, androgen - dependent CWR22 xenograft | Express PSA | Express, with weak stimulation by dihydrotestosterone | The transcription of mRNA for PSMA is almost two - fold greater than that for PSA | Partially androgen - dependent |
| DU145 | Brain metastasis of prostate cancer | None or very low expression | None or extremely low expression | Not expressed | Independent |
| RWPE - 1 | Isolated from the peripheral zone of the prostate of a 54 - year - old white male patient without evidence of prostate cancer | Very low or nonexistent under standard culture conditions, can secrete PSA in three - dimensional Matrigel culture when exposed to androgen | Expressed, relatively low level | Generally considered to be expressed at a low level like normal prostate epithelial cells | Androgen - responsive, shows a weak response to androgen stimulation |

**References:​**

Horoszewicz JS, Leong SS, Kawinski E, et al. LNCaP model of human prostatic carcinoma. Cancer Res. 1983;43(11 Suppl):5070s - 5078s.​

Kaighn ME, Narayan KS, Ohnuki Y, Lechner JF, Jones PA. Establishment and characterization of a human prostatic carcinoma cell line (PC - 3). Invest Urol. 1979;17(1):16 - 23.​

Wu HC, Hsieh JT, Gleave ME, et al. Derivation of androgen - independent human LNCaP prostatic cancer cell sublines: role of bone stromal cells. Int J Cancer. 1994;57(3):406 - 412.​

Kantoff PW, Kao CS, Hong SH, et al. Androgen - independent growth of a human prostate cancer xenograft (CWR22) after androgen withdrawal. Cancer Res. 1994;54(19):5070 - 5074.​

Stone KR, Mickey DD, Wunderli H, et al. Isolation of a human prostate carcinoma cell line (DU 145). Int J Cancer. 1978;21(2):274 - 281.​

Thomson TM, Maitland NJ. RWPE - 1: a new normal human prostate epithelial cell line. In Vitro Cell Dev Biol Anim. 1996;32(11):696 - 700.
